# Comparative Study on the Neuroprotective Effects of Perindopril and Benazepril in Experimentally-induced Chronic Mild Stress in Rats

**DOI:** 10.1007/s11481-025-10244-z

**Published:** 2025-10-09

**Authors:** Alaa M. Badawy, Amany M. Gad, Amany E. Abdel-Maged, Haidy E. Michel, Reem N. El-Naga, Samar S. Azab

**Affiliations:** 1 Egyptian Drug Authority (EDA), Cairo, Egypt; 2 Department of Pharmacology and Toxicology, Faculty of Pharmacy, East Kantara Branch, Sinai University, Ismailia, Egypt; 3 Department of Pharmacology and Toxicology, Faculty of Pharmacy, Ain Shams University, Cairo, Egypt

**Keywords:** Depression, Chronic unpredictable mild stress, ACE inhibitors, Perindopril, Benazepril, Antidepressant effects, Neuroinflammation, RAAS pathway

## Abstract

Depression remains a major global health issue, characterized by inadequate response rates to conventional antidepressant therapies. This highlights a critical need for novel treatment strategies. Our study investigated the antidepressant effects of benazepril, a non-centrally acting angiotensin-converting enzyme (ACE) inhibitor, and compared it with perindopril, a centrally acting ACE inhibitor. We utilized a rat model of depression induced by chronic unpredictable mild stress (CUMS) to evaluate their efficacy. The CUMS protocol effectively caused several depression-like behaviors and impaired neurobehavioral functions in the rats. Analysis of brain tissues from these animals revealed several key pathological hallmarks: diminished monoamine neurotransmitter levels, heightened oxidative stress, robust inflammatory responses, and increased apoptotic processes. Our findings demonstrated that both perindopril and benazepril significantly reversed these CUMS-induced deficits. Specifically, both ACE inhibitors exhibited potent antioxidant, anti-inflammatory, and anti-apoptotic properties. This was coupled with their effective inhibition of the renin-angiotensin-aldosterone system (RAAS) signaling pathway, a mechanism known to be implicated in stress responses and mood disorders. Notably, while many ACE inhibitors have been extensively studied for their central effects, research on benazepril’s direct effects within the brain or central nervous system in rats is notably limited. This study is among the first to highlight the antidepressant potential of benazepril, a non-centrally acting ACE inhibitor, and provides novel insights into its comparative efficacy against perindopril. These results collectively emphasize the broader therapeutic potential of ACE inhibitors in treating depression and underscore the need for further research to fully explore their underlying mechanisms and diverse applications in psychiatric disorders.

**Graphical Abstract:**

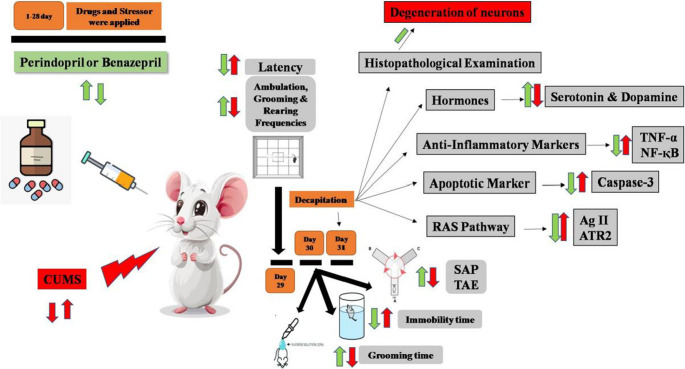

**Supplementary Information:**

The online version contains supplementary material available at 10.1007/s11481-025-10244-z.

## Highlights

CUMS induced diminished monoamine levels, heightened oxidative stress, inflammatory responses, and apoptotic processes have been observed in the rat brain.

Both perindopril and benazepril significantly reversed these depressive-like behaviors and impaired neurobehavioral functions.

Both perindopril and benazepril demonstrated potent antioxidant, anti-inflammatory, and anti-apoptotic properties while inhibiting the renin-angiotensin-aldosterone system signaling.

**Supplementary Information:**

The online version contains supplementary material available at 10.1007/s11481-025-10244-z.

## Introduction

Globally, depressive disorders are a growing public health issue, impacting approximately 300 million individuals (Herrman et al. 2019). It is characterized by persistent feelings of sadness and disinterest in activities that were once enjoyed. Depression can disrupt sleep patterns and appetite, and in severe cases, may lead to suicide. This mental illness is influenced by various factors such as genetics, environment (e.g., poverty, traumatic life events, childhood abuse), psychology (e.g., cognitive patterns), and biology (e.g., inflammation, neurotransmitter pathways), with exposure to stress being a recognized risk factor (Ma et al. 2021).

Depression is strongly linked to an increased risk of serious health conditions, including stroke, dementia, and hypertension. For example, depression can persist for six months following a stroke, which not only impedes neurological recovery but also increases the likelihood of another stroke and mortality (Dong et al. 2021; Nickel and Thomalla 2017). Furthermore, depression is associated with a heightened risk of developing dementia and Alzheimer’s disease, sometimes surpassing traditional risk factors like smoking, hyperglycemia, and hypercholesterolemia (Alexopoulos 2019; Diniz 2018). Additionally, depression raises the risk of hypertension by 42% (Meng et al. 2012). Given these clinically significant findings, there is a pressing need for improved strategies and innovative drug targets to effectively address depressive disorders (Hackett and Anderson 2005; Herring et al. 2014).

Hypertension is a growing global health crisis, affecting over one billion people and driving cardiovascular diseases, stroke, and premature deaths (Kario et al. 2024; World Health Organization 2023). Individuals with cardiovascular and cerebrovascular diseases, as well as those diagnosed with hypertension, often experience depression, which can have negative implications on important clinical outcomes. Moreover, the prevalence of major depression among individuals with hypertension, post-myocardial infarction, and post-stroke is approximately 30% for each of these conditions (Doyle et al. 2015; Li et al. 2015; Meijer et al. 2013).

Beyond its well-established function in the regulation of peripheral cardiovascular mechanisms, a comprehensive and operationally significant renin-angiotensin-aldosterone system (RAAS) is intrinsically present within the critical brain regions for mood regulation, cognitive function, and stress responses (Ali et al. 2024; Gong, 2022; Guimond and Gallo-Payet 2012). Dysregulation of this central RAAS, particularly characterized by an excessive activation of the Ang II-AT1 receptor pathway, is increasingly recognized as a pivotal factor in the pathophysiological mechanisms underlying depression. Such hyperactivity may result in augmented neuroinflammation, increased oxidative stress, impaired neurogenesis, and alterations in neurotransmitter systems – processes profoundly implicated in the development and progression of depressive states (Miranda et al. 2024; Gong 2022; Vian et al. 2017).

The regulation of water and electrolytes, as well as blood pressure, is managed by the renin-angiotensin system (RAS), but foundational research has extensively demonstrated its significant involvement in neurological conditions, emotional disturbances, and hypertension (Karwowska-Polecka et al. 2002a; Li et al. 2015; Zaman et al. 2002). The RAS operates through an intricate balance between its classical(ACE/Ang II/AT1 receptor) and non-classical (ACE2/Ang-(1–7)/MasR) pathways, which maintain equilibrium under normal physiological conditions. Crucially, activation of the ACE2/Ang-(1–7) Mas receptor system has been found to mitigate the development of mood disorders, Conversely, excessive expression of the ACE/Ang II/AT1 receptor pathway intensifies feelings of anxiety and depression. There is evidence indicating that heightened RAS activity is associated with anxiety and depression, potentially linked to triggering neuroinflammation and oxidative stress (Welcome and Mastorakis 2020).

The renin enzyme breaks down angiotensinogen into angiotensin I (Ang I) in both central and non-central regions of RAS (Miller and Arnold 2019).Beyond the peripheral RAS, the central nervous system (CNS) possesses its own receptors that contribute to neurodegeneration through processes like oxidative stress, neuroinflammation, and neuronal death (Abiodun and Ola 2020; Xu et al. 2021). The brain possesses its own intrinsic Renin-Angiotensin System (RAS), separate from the peripheral system, identified in 1971 (Ganten et al. 1971). Within this system, angiotensin II (Ang II) is formed when angiotensin-converting enzyme (ACE) converts angiotensin I. Ang II primarily acts through its type 1 (AT1) and type 2 (AT2) receptors, which are G-protein coupled receptors mediating various physiological effects (Lee 1981, p. 199).

Strong clinical evidence supports the direct relevance of RAAS dysregulation in human depression, suggesting that depressed patients, particularly those with comorbid hypertension, exhibit altered RAAS activity profiles (Haefner et al. 2013). This link is further supported by extensive epidemiological studies examining the impact of antihypertensive drugs on depression risk (Kessing et al. 2020).Moreover, some classes of antihypertensive drugs, such as ACE inhibitors, are linked to a lower risk of developing depression. According to these human population studies, RAAS modulation may have a direct or indirect impact on mood regulation, which may lead to new ways of treating depression (Miranda et al. 2024; Zaman et al. 2002).

Preclinical studies in a variety of animal models of anxiety and depression are increasingly supporting these clinical correlations. Proven antidepressant and anxiolytic effects have been demonstrated for RAAS inhibitors. Blocking AT1 receptors consistently increases neuroplasticity, reduces oxidative stress and neuroinflammation in impacted brain regions, and reverses stress-induced depressive behaviors (Lenart et al. 2019). Specific AT1 receptor blockers like losartan (Diniz et al. 2018), valsartan (Ping et al. 2014), irbesartan (Ayyub et al. 2017), and telmisartan (Li et al. 2018) have all shown promise in various animal models of depression and anxiety, influencing mechanisms such as neurogenesis, BDNF expression, and cerebral activity.

The neuroprotective and mood-enhancing effects of ACE inhibitors have been well-documented. Luo et al. (2020a, b) demonstrated that the ACE inhibitor captopril rapidly ameliorated depressive-like behaviors in mice via bradykinin-dependent activation of the mTORC1 pathway, revealing a novel mechanism. Additionally, Balogh et al. (2020) demonstrated the antidepressant effects of the ACE inhibitors, enalapril and ramipril, in models of diabetes-associated depression. Coşkun et al. (2010) also pointed out the neuroprotective effects of ACE inhibitors, including perindopril, ramipril, and captopril, in cerebral ischemia—processes that are commonly linked to the pathophysiology of depression. These findings provide convincing pharmacological evidence and mechanistic precedent for examining the therapeutic potential of ACE inhibitors, such as benazepril and perindopril, in reducing the behavioral and neurobiological impairments linked to depression.

Given its involvement in neurodegenerative pathways, inhibitors of the RAS that can penetrate the blood-brain barrier (BBB) and impede neurodegenerative pathways may offer neuroprotective effects in brain disorders, such as Alzheimer’s disease (Lee et al. 2023). Conditions like diabetes and neuropsychiatric disorders, including depression and Alzheimer’s disease, have been associated with the excessive activation of the RAS, which is often connected with neurodegeneration and neuroinflammation (Gebre et al. 2018; Vargas et al. 2012; Yagi et al. 2013).

Stress has been widely recognized as a contributing factor to the development of major depression. Accordingly, the chronic unpredictable mild stress (CUMS) rat model has been widely used to mimic the onset and progression of clinical depression (American Psychiatric Association 2000; Willner 1997). This experimental model has become a common tool for studying depression-related issues because it can replicate different aspects of the disorder (Hu et al. 2017). Notably, Chronic stress is known to interfere with the adaptability, characteristics, and functioning of microglial cells, which are considered essential components of depression-related brain pathology (Yirmiya et al. 2015).

The Chronic Unpredictable Mild Stress (CUMS) model entails subjecting rats to a variety of mild and unpredictable stressors and has been widely utilized in the assessment of depressive behaviors. Recent research affirms that the CUMS model consistently triggers behavioral changes similar to those seen in clinical depression, such as reduced consumption of sucrose, loss of interest in pleasurable activities, disrupted sleep patterns, alteration in body weight, decreased physical activity, deterioration in fur quality, reduced response to rewards, and heightened aggression (Liu et al. 2014).

Angiotensin-converting enzyme inhibitors (ACEIs) differ significantly in their ability to cross the blood-brain barrier (BBB). Some ACEIs, including captopril, fosinopril, lisinopril, perindopril, ramipril, and trandolapril, are considered centrally active because they can penetrate the brain. In contrast, others, including benazepril, enalapril, moexipril, and quinapril, are non-centrally active, generally not crossing into the brain (Johnston et al. 1988; Tan et al. 2005). Early clinical observations from the 1980 s in Europe and the United States have already associated ACEIs, such as captopril and enalapril, with a swift improvement in patients’ depressive symptoms (Karwowska-Polecka et al. 2002b).

Perindopril, a long-acting ACEI globally known for effective antihypertensive properties, has gained recognition for its high tissue ACE affinity (Yamada et al. 2011). Similarly, benazepril, another ACEI, has been utilized to manage chronic renal failure, hypertension, and congestive heart failure (Chan et al. 1994; King et al. 2017a). However, while perindopril has received considerable attention for its anti-inflammatory and neuroprotective properties, research on benazepril’s specific effects on depression or brain function in rats is limited.

Accordingly, the present study aimed to examine and compare the potential neuroprotective properties of both perindopril and benazepril within the chronic unpredictable mild stress (CUMS)rat model of depression. This study is particularly novel in its direct head-to-head comparison of a centrally acting ACE inhibitor (perindopril) with anon-centrally/peripherally acting one (benazepril) to highlight their differential effects on depression-related pathology. Furthermore, this study aimed to explore the possible mechanisms behind this protective effect, focusing on how they affect apoptosis and inflammation. The study also sought to as certain if perindopril or benazepril, as ACEIs, may trigger signaling in rats’ central renin-angiotensin-aldosterone system (RAAS) pathway.

## Materials and Methods

### Animals

The National Organization for Drug Control and Research (NODCAR) breeding colony provided the male Sprague-Dawley rats, which weighed between 180 and 200 g. Before the experiment began, the rats were given a week to get used to their new surroundings in the lab room under normal housing conditions. They were kept on a 12-hour light/dark cycle at 22 ± 2 °C and 40–60% relative humidity. Water and food pellets were freely available to them. To lessen the suffering of the animals, they were treated tenderly. Additionally, the experiments were carried out following approval by the Ain Shams University Faculty of Pharmacy Ethics Committee (No. 252).

### Materials

Saline was used to dissolve benazepril (DBK Pharmaceutical Industries, Egypt) and perindopril arginine (Servier; Coversyl^®^). The medications were freshly made. Every additional chemical employed in this investigation was of the greatest analytical level and purity that could be purchased.

### Drug and Dose Selection

Our study’s innovative approach lies in the direct comparison of benazepril, a non-centrally acting angiotensin-converting enzyme inhibitor (ACEI), with perindopril, a known centrally acting ACEI. This head-to-head comparison to assess their antidepressant potential within a rat Chronic Unpredictable Mild Stress (CUMS) model of depression is, to our knowledge, the first of its kind. It specifically contrasts their known primary actions—peripheral versus central—to shed light on the mechanisms underlying their effects.

The rationale for selecting these drugs is rooted in the concept of drug repositioning and the growing body of clinical and preclinical evidence implicating the Renin-Angiotensin-Aldosterone System (RAAS) in the pathophysiology of depression. While numerous ACE inhibitors and Angiotensin Receptor Blockers (ARBs) have been explored for their effects on the central nervous system (CNS) (as extensively reviewed by (Ali et al. 2024; Miranda et al. 2024; Gong 2022; Zaman et al. 2002), benazepril stands out due to a notable scarcity of research specifically investigating its effects within the brain or CNS. Most existing studies on benazepril focus primarily on its peripheral cardiovascular and renal actions (Charan Sahoo et al. 2009; King et al. 2017b; Li et al. 2010; Tang et al. 2015). This significant gap in understanding its cerebral potential made exploring its role in brain disorders, particularly CUMS-induced depression, a compelling endeavor.

The idea of drug repositioning and the increasing amount of preclinical and clinical data linking the Renin-Angiotensin-Aldosterone System (RAAS) to the pathophysiology of depression serve as the foundation for the reasoning for the choice of these medications. While numerous ACE inhibitors and Angiotensin Receptor Blockers (ARBs) have been explored for their effects on the central nervous system (CNS), as reviewed in detail in recent and old studies (Ali et al. 2024; Miranda et al. 2024; Gong 2022; Zaman et al. 2002). However, benazepril stands out due to a notable scarcity of research specifically examining its effects within the brain or CNS. Most existing studies on benazepril focus primarily on its peripheral cardiovascular and renal actions (Charan Sahoo et al. 2009; King et al. 2017b; Li et al. 2010; Tang et al. 2015). This significant gap in understanding its cerebral potential made exploring its role in brain disorders, particularly CUMS-induced depression, a compelling endeavor.

On the other hand, perindopril was selected as a comparator due to its well-established ability to effectively penetrate the CNS. A vast body of research supports its neuroprotective and advantageous benefits in various neurological conditions. Existing literature shows perindopril’s efficacy in models of Alzheimer’s disease (Hou et al. 2008; Messiha et al. 2020), Parkinson’s disease (Kurosaki et al. 2004), and dementia (Alzahrani et al. 2020). It has also demonstrated neuroprotective benefits against inflammation-induced injury (Ali et al. 2016; Şen et al. 2022), and broadly as an ACEI in cerebral ischemia (Jawaid et al. 2015). This rich background of known central activity for perindopril made it an ideal drug to compare against benazepril’s largely unexplored CNS profile, allowing for a clearer differentiation of potential central versus peripheral mechanisms in alleviating depressive symptoms.

Benazepril and perindopril were both prescribed at a constant dose of 10 mg/kg/day after a comprehensive analysis of the preclinical literature. In numerous rat and mouse models of neurological and systemic disorders, thisdosage has been widely demonstrated in earlier studies to effectively elicit significant antioxidant, anti-inflammatory, and neuroprotective effects. It has been demonstrated that this dosage of benazepril reduces organ damage, such as cardiotoxicity and nephrotoxicity (Jin et al. 2014; Li et al. 2010; Nazmi et al. 2016). Furthermore, it improves vascular relaxation and efficiently lowers oxidative stress in hypertensive models (Singh et al. 2008; Tschudi et al. 1994). Similarly, the 10 mg/kg/day dose of perindopril has a documented record of protective actions in preclinical studies. It demonstrates significant benefits on pulmonary vascular structure and function in hypertensive rats (Jeffery and Wanstall 1999) and exhibits potent antihypertensive actions comparable to other ACE inhibitors (Unger et al. 1986).

Importantly, in models of inflammation and brain injury, this dose has consistently produced neuroprotective and anti-inflammatory effects (Ali et al. 2016; Şen et al. 2022). This guarantees that any observed antidepressant-like actions in the CUMS model could be confidently attributed to their established antioxidant, anti-inflammatory, and protective properties, which are extremely relevant to the pathophysiology of depression.

### Experimental Design

Forty-eight mature male Sprague-Dawley rats weighing 180–200 g participated in this investigation. Rats were segregated into six groups at random, with eight rats each: Rats in **Group I**, the saline control group, were given only vehicle. Rats in **groups II and III** were the drug control groups; rats in these groups were given only medicines (either perindopril or benazepril, 10 mg/kg/day, respectively) orally by gavage. Rats in **group IV **acted as the CUMS group, while rats in **groups V and VI** were the therapy groups; for the perindopril and the benazepril-treated groups, depressed rats were given a single oral gavage dosage of benazepril and perindopril (10 mg/kg/day, respectively) (Li et al. 2010; Unger et al. 1986). Behavioral tests were performed at the conclusion of the experiment. Rats were then decapitated, and their brains were removed. The whole brain from three rats per group were preserved in 10% formalin for histological analysis. From other rats, the cortices and hippocampi of the brain, were collected and stored at −80 °C until biochemical examinations.

**Fig. 1:**
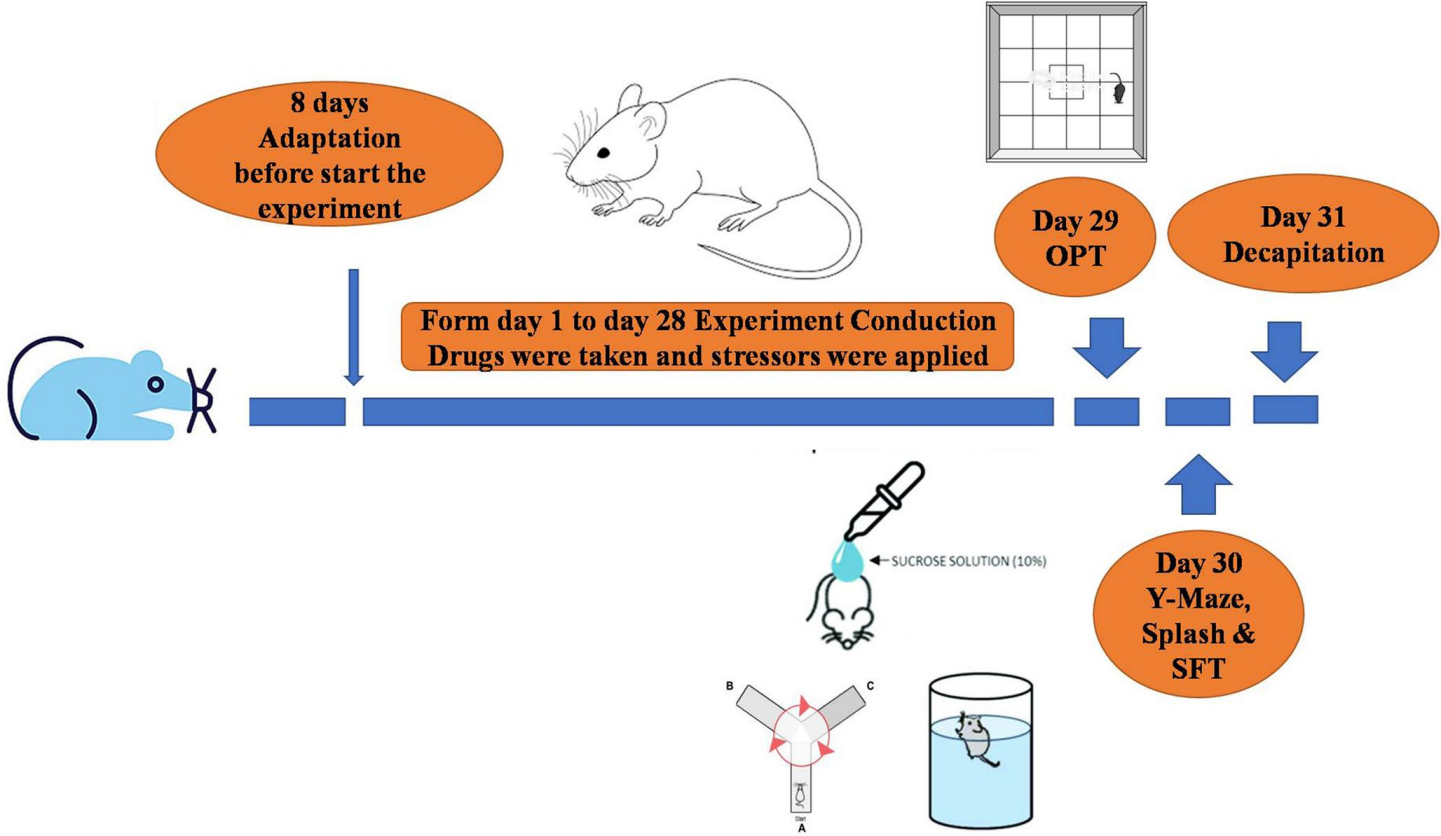
The timeline of the experimental procedure includes a 4-week period of conducting the experiment, which involves various behavioral tests such as the open field test (OPT), Y Maze, Splash test, and forced swimming test (FST), concluding with decapitation

The present investigation utilized a modified unexpected CMS procedure (Goshen et al. 2008; Lu et al. 2014; Peng et al. 2012). This model was originally created by Willner (1997). It mimics how clinical depression develops and progresses, including decreased reactivity to rewarding stimuli, decreased intake of sucrose, decreased locomotor activity, altered weight gain, and physical status deterioration. Throughout the four-week experiment, rats were continuously exposed to a variety of random, low-intensity stressors as follows:15 min of forced swim in 25-°C water, 18 h of wet bedding, 18 h of food and water deprivation, 18 h of cage tilting (at 45°), 3 h of tube restraint, 24 h of constant light, and 24 h of light/dark cycle reversal (Fig. 1). To prevent the rats from memorizing or anticipating the stressors, which are listed in Table 1, they were applied at irregular intervals throughout the day.

**Table 1:** The applied stressors were used to inducedepression in experimental rats using the CUMS model

Day week	Day 1	Day 2	Day 3	Day 4	Day 5	Day 6	Day 7
Week (1)	Imprisonment in a tube (3 h)	Damp bedding and titled cage (45°) (18 h)	Imprisonment in a tube (3 h)	Imprisonment in a tube (3 h)	Swimming at (25 °C) (15 min)	Damp bedding and titled cage (45°) (18 h)	Continuous light on (24 h)
Week (2)	Imprisonment in a tube (3 h)	Damp bedding and titled cage (45°) (18 h) + food or water deprivation (18 h)	Imprisonment in a tube (3 h) + food and water deprivation (18 h)	Imprisonment in a tube (3 h) + titled cage (45°) (18 h)	Imprisonment in a tube (3 h) + Damp bedding (18 h)	Inversion of light\dark cycle (24 h) + food and water deprivation (18 h)	Damp bedding and titled cage (45°) (18 h) +food or water deprivation (18 h)
Week (3)	Swimming at (25 °C) (15 min)	Damp bedding and titled cage (45°) (18 h)	Imprisonment in a tube (3 h) + Continuous light on (24 h)	Imprisonment in a tube (3 h) + food or water deprivation (18 h)	Imprisonment in a tube (3 h) + Damp bedding and titled cage (18 h)	Imprisonment in a tube (3 h) + food or water deprivation (18 h)	Imprisonment in a tube (3 h) + Continuous light on (24 h)
Week (4)	Swimming at (25 °C) (15 min)	Damp bedding and titled cage (45°) (18 h)	Imprisonment in a tube (3 h) + food or water deprivation (18 h)	Swimming at (25 °C) (15 min)	Imprisonment in a tube (3 h)	Damp bedding and titled cage (45°) (18 h)	Imprisonment in a tube (3 h)

### Behavioral Tests

#### Open field Test

The motor activity was evaluated using the open field test (Costall et al. 1988). It was carried out following previous studies (Abdrabou et al. 2025; Esmail et al. 2024; Kamal et al. 2022). Each rat was positioned in the middle square and watched for 5 min (Vorhees 1974). Following each experiment, 5% ethanol was used to thoroughly clean the walls and floor. During the five minutes, the number of squares each rat crossed (Zbinden 1981), the time each rat took to begin moving/the latency to initiate movement (Van den Buuse and de Jong 1989), the times each rat stood on its hind limbs (Van den Buuse and de Jong 1989), and the number of grooming bouts were noted.

#### Y-Maze Test

Three identical opaque arms (50 cm in length, 20 cm in height, and 10 cm in breadth) that are labeled arm A, B, or C and intersect at a 120° angle, form the device, a white wooden maze. When all four paws were inside the arm, a manual entry was considered valid. If the rat consecutively entered each of the three arms, the spontaneous alternation was scored. In accordance with (Holcomb et al. 1998; Hooper et al. 1996; Kamal et al. 2022; Shalaby et al. 2019), the test was conducted and scored.

#### Forced Swimming Test

The effectiveness of putative antidepressant medications is evaluated using the forced swimming test (Kirby and Lucki 1997). Water was added to the 40 cm high by 22 cm wide cylinder tank until it reached a depth of 25 cm at room temperature (25 °C) (Porsolt et al. 1977, 1978). Both the immobility time, defined as passively floating with minimal movements to keep its head above water for 5 min, and the struggling time, defined as active attempts to escape or climb the tank walls, were noted (Volosin et al. 1988).To prevent ambiguous results, the water in the cylinder was changed after every trial (Einat et al. 1999).A pre-exposure swim, 24 h before the test session, was conducted to discern antidepressant-like activity (Slattery and Cryan 2012).

#### Splash Test

With a few minor adjustments, this test was conducted as previously described (Isingrini et al. 2010). It involves placing each rat separately in a clear plexiglas cage (9 × 7 × 11 cm^3^) and squirting a 10% sucrose solution onto its dorsal coat. The viscosity of the sucrose solution causes the rat fur to get dirty, which prompts the animals to begin grooming. Following the application of the sucrose solution, grooming time was manually recorded for five minutes as a measure of motivational behavior and self-care, considered analogous to the apathetic symptoms of depression (Willner 2005). To conceal animal traces, the equipment was cleaned with a 5% ethanol solution after each trial.

### Histopathological Examination

Rats in various groups had their brains harvested, and the samples were processed in accordance with (Bancroft and Gamble 2008). A pathologist who was not aware of the identity of the material conducted a histopathological investigation to prevent bias. Nissl stain was utilized to assess neuron vitality (Bancroft and Gamble 2008).

### Biochemical Parameters

#### The Evaluation of the Levels of Brain Neurotransmitters

Rat-specific enzyme-linked immunosorbent assay (ELISA) kits, which were acquired from BT LAB, Shanghai Korain Biotech Co., Ltd., China, were used to determine the dopamine and serotonin (5-HT) content in the homogenized tissues of the cortex and hippocampus. The kits had the respective Cat. Nos. EA0111Ra and E0866Ra for dopamine and serotonin. Neurotransmitter concentrations were reported as ng/L for dopamine and ng/mL for serotonin.

#### The Evaluation of Inflammatory Biomarkers

Using a rat-specific TNF-α ELISA kit (Cat. No. E0764Ra) from BT LAB, Shanghai Korain Biotech Co., Ltd., China, tumor necrosis factor-alpha (TNF-α) was quantified in the cortex and hippocampus tissue homogenates and expressed as ng/L. Immunohistochemistry was used to measure the expression of nuclear factor kappa B (NF-κB) in brain regions. Following the manufacturer’s instructions, the immunohistochemical detection of NF-κB in the brain was carried out. For immunological staining, brain slices with a thickness of 5 μm were sectioned and placed on adhesive slides. After rehydrating tissue slides with distilled water and performing heat-mediated epitope retrieval, they were incubated with the primary antibody anti-NF-κB (Santa Cruz Biotechnology, Inc., Cat. No. sc-8008) for 1 h at room temperature in a humid environment. Following washing, hydrogen peroxide was used to block endogenous peroxidase activity in tissue sections. To construct the reaction, an HRP-labeled detection kit (BioSB, USA) was used. By avoiding incubation with the primary antibody, negative control slides were produced. Immunohistochemical staining was quantified semi-quantitatively using ImageJ software (v.1.50i, NIH, USA) by measuring integrated optical density (IOD) within consistent Regions of Interest (ROI) after thresholding (Crowe and Yue 2019).

#### The Evaluation of Apoptotic Biomarkers

Immunohistochemical detection of Caspase-3 in the brain was carried out as described previously, using primary anti-Caspase-3 (Santa Cruz biotechnology, Inc., Cat. No.sc-7272) in accordance with the manufacturer’s instructions. By avoiding incubation with the primary antibody, negative control slides were produced. Immunohistochemical staining was quantified semi-quantitatively using ImageJ software (v.1.50i, NIH, USA) by measuring integrated optical density (IOD) within consistent Regions of Interest (ROI) after thresholding (Crowe and Yue 2019).

#### Western Blot of Angiotensin II (Ag II) and Angiotensin Type 2 Receptor(ATR2) in Brain Tissues

The western blot technique was used to measure the amounts of Angiotensin II (Ag II) and Angiotensin Type 2 Receptor (ATR2) proteins in the cortex and hippocampus regions, as previously described by Elhadary et al. (2021).

Proteins (30 µg) were loaded and separated using sodium dodecyl sulfate–polyacrylamide gel electrophoresis (SDS-PAGE) in accordance with (Bamdad et al. 2009). Following separation, proteins were transferred to a Hybond™ nylon membrane (GE Healthcare) using a TE62 Standard Transfer Tank with Cooling Chamber (Hoefer Inc.). The membranes were then incubated with primary antibodies: anti-ANGIOTENSIN II (Catalog No. ABIN74454423, antibodies-online.com, USA), anti-Angiotensin type 2 receptor (EPR3876, Abcam, UK), or **anti-β-Actin antibody [mAbcam 8224] - BSA and Azide free (Abcam**,** UK)**. This was followed by incubation with appropriate horseradish peroxidase (HRP)-conjugated secondary antibodies (antibody concentration: 0.1–0.5 µg/mL). The resulting band intensity was quantified using a Gel documentation system (Geldoc-it, UVP, England), and the data were analyzed using TotalLab analysis software (Ver.1.0.1).

### Statistical Analysis

Mean ± standard deviation (SD) was used to express the data. The Bonferroni multiple comparison test and Two-way ANOVA were used to assess the statistical significance of the means of the various groups. P-values > 0.05 were regarded as statistically significant. GraphPad Prism program (Inc. V5, San Diego, CA, USA) was used to display the results.

## Results

### Behavioral Outcomes in Rats Exposed to the CUMS Model

#### The Open-field Test

**Fig. 2:**
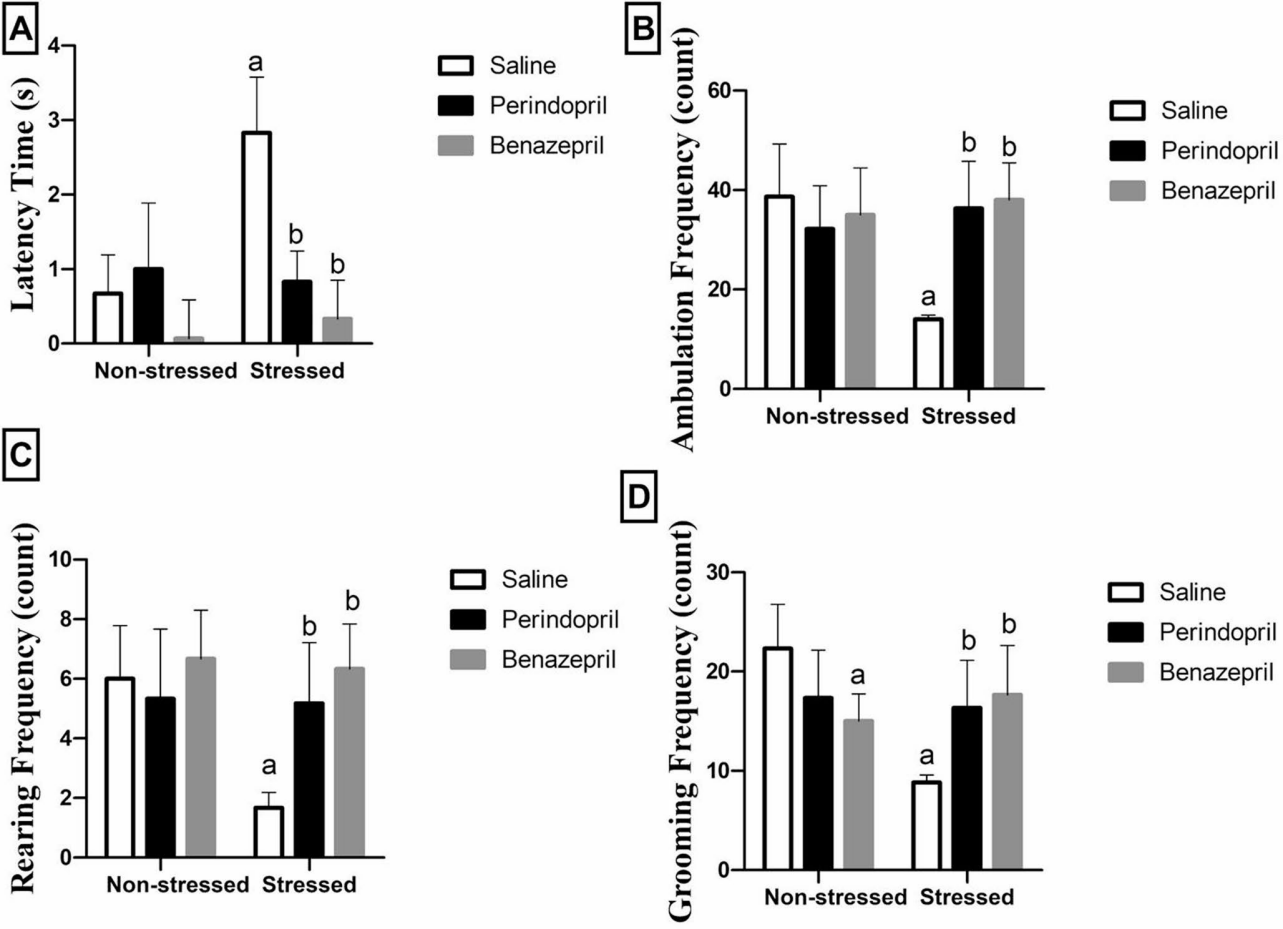
The effects of perindopril and benazepril) on the behavioral tests; Latency time (**A**), Ambulation frequency (**B**), Rearingfrequency (**C**), GroomingFrequency (**D**) in rats that were exposed to the mild, prolonged, unpredictable stress. The means ± standard deviation (*n* = 6) are used to display the data. Bonferroni’s multiple comparison test was used after a two-way ANOVA for statistical analysis. At a significance level of *p* < 0.05, Significant differences from non-stressed saline group (saline control group), Stressed saline group (CUMS group), and Stressed perindopril-treated group (perindopril-treated group)(10 mg/kg) are indicated by labels a, b, and c, respectively. The following is the acronyms: CUMS for chronic unpredictable mild stress

Two-way ANOVA showed that the stress protocol and the different treatments (saline, perindopril and benazepril) had significant main effect on open field behavioral parameters: Latency time (stress: F(1,30) = 13.05, *p* = 0.0011; different treatments: F(2,30) = 18.60, *p* < 0.0001; stress by treatment: F(2,30) = 11.84, *p* = 0.0002), Ambulation Frequency (stress: F(1,30) = 4.33, *p* = 0.0461; different treatments: F(2,30) = 4.83, *p* = 0.0152; stress by treatment: F(2,30) = 11.28, *p* = 0.0002), Rearing frequency (stress: F(1,30) = 7.755, *p* = 0.0092; different treatments: F(2,30) = 7.092, *p* = 0.0030; stress by treatment: F(2,30) = 5.542, *p* = 0.0089). On the other hand, Grooming frequency statistical analysis showed that the stress protocol had significant main effect (stress: F(1,30) = 8.461, *p* = 0.0068; stress by treatment: F(2,30) = 13.03, *p* < 0.0001), without any significant main effect for different treatments **(**Fig. 2A-D**).**

Post-hoc Bonferroni tests revealed that both benazepril and perindopril exhibited comparable efficacy in ameliorating CUMS-induced behavioral deficits, demonstrating similar antidepressant-like effects. Specifically, the CUMS group displayed significant behavioral alterations characteristic of a depressive-like state. Compared to the saline control group, the CUMS group showed a significant increase in latency time (322.38%) and a notable reduction in ambulation (63.80%), grooming frequencies (60.46%), and rearing (72.17%).

Both drug treatments effectively reversed these impairments induced by CUMS. The perindopril-treated group demonstrated a significant reduction in latency time (70.67%) and a significant increase in ambulation (159.50%), grooming frequencies (84.94%), and rearing (209.58%) compared to the CUMS group. Similarly, the benazepril-treated group also exhibited a significant reduction in latency time (88.34%) and a significant rise in ambulation (171.43%), grooming frequencies (100.11%), and rearing (279.04%) relative to the CUMS group.

Crucially, our statistical analysis confirmed no significant differences between the benazepril and perindopril-treated groups across any of these behavioral parameters, underscoring their comparable therapeutic impact.There is a significant difference between benazepril only group and saline control group in grooming frequency.

#### The Y-Maze Test

**Fig. 3:**
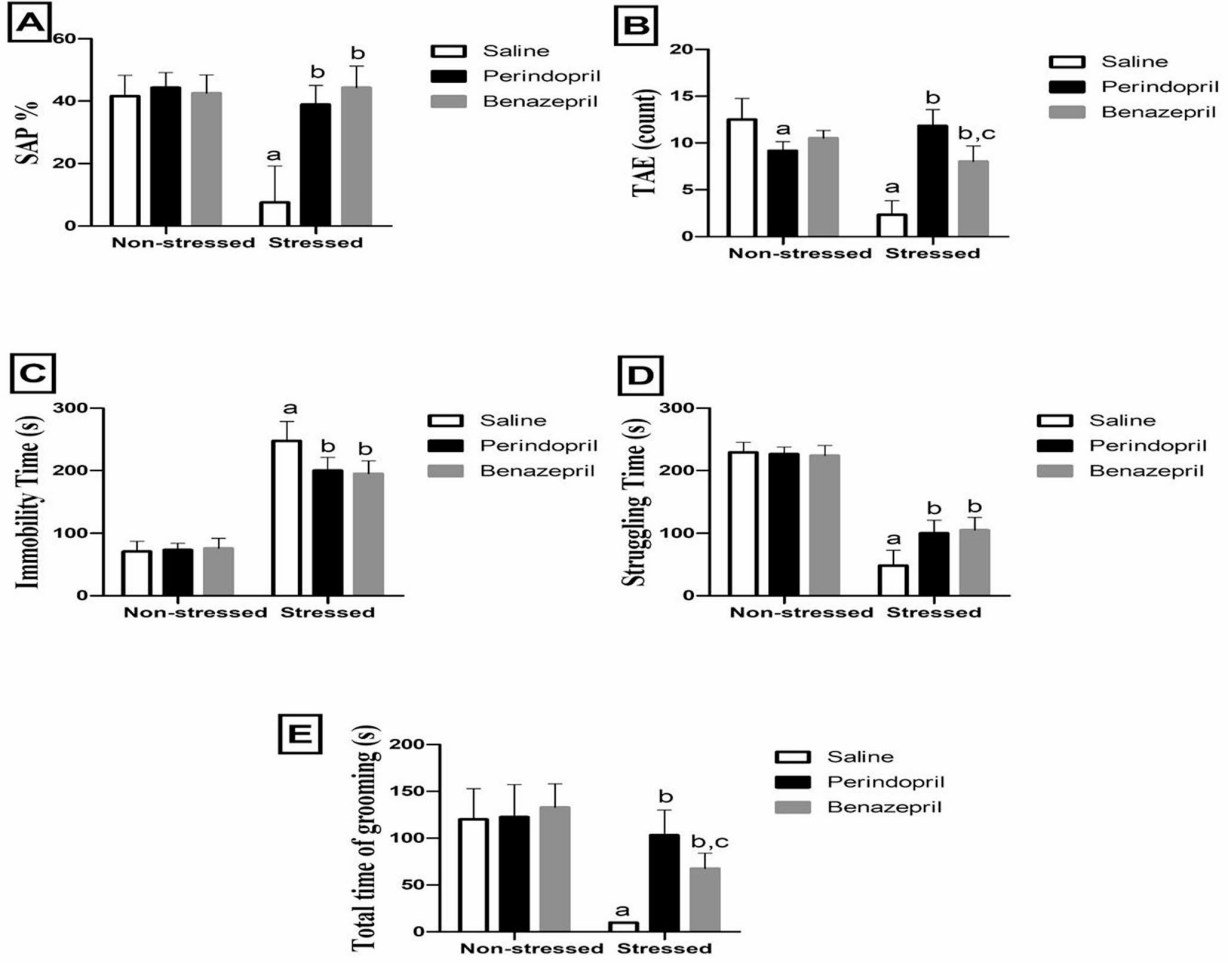
The effects of perindopril and benazepril on the behavioral tests; spontaneous percentage of alternation (SAP) (**A**), Total Arm Entries count (TAE) (**B**), Immobility time (**C**), Struggling time (**D**) and Total time of grooming (**E**) in rats that were exposed to the mild, prolonged, unpredictable stress. The means ± standard deviation (*n* = 6)are used to display the data. Bonferroni’s multiple comparison test was used after a two-way ANOVA for statistical analysis. At a significance level of *p* < 0.05, Significant differences from non-stressed saline group (saline control group), Stressed saline group (CUMS group), and Stressed perindopril-treated group (perindopril-treated group)(10 mg/kg) are indicated by labels a, b, and c, respectively. The following are the acronyms: TAE stands for total arm entries, SAP for spontaneous percentage of alternation, and CUMS for chronic unpredictable mild stress

Short-term spatial memory function was evaluated using the Y-maze test (Fig. 3A&B). Two-way ANOVA showed that the stress protocol and the different treatments (saline, perindopril and benazepril) had significant main effect on Y-Maze parameters such as : SAP (stress: F(1,30) = 26.03, *p* < 0.0001; different treatments: F(2,30) = 23.96, *p* < 0.0001; stress by treatment: F(2,30) = 19.91, *p* < 0.0001), and TAE (stress: F(1,30) = 40.62, *p* < 0.0001; different treatments: F(2,30) = 11.71, *p* = 0.0002; stress by treatment: F(2,30) = 50.69, *p* < 0.0001).Our findings indicate that while both perindopril and benazepril effectively ameliorated CUMS-induced cognitive deficits, perindopril demonstrated a more robust impact on overall exploratory activity.

Specifically, the CUMS group demonstrated a significant decline in cognitive function compared to the saline control group, as evidenced by the decreased spontaneous percentage of alternation (SAP) and total arm entries (TAE) by 81.96% and 81.36%, respectively.

Conversely, both drug treatments significantly improved cognitive function relative to the CUMS group. Perindopril-treated rats showed a notable increase in SAP and TAE by 418.93% and 407.72%, respectively. Similarly, benazepril-treated rats also exhibited significant improvements, with SAP increasing by 476.27% and TAE by 243.35%. However, we did notice a curious distinction in their exploratory behavior: the benazepril group had a significantly lower TAE (32.38% reduction) compared to the perindopril group. This hints at a different effect on general activity, even though both drugs seemed equally effectiveat improving spatial alternation. On the other hand, there is a significant difference between perindoprilonly group and saline control group in TAE.

####  The Forced Swimming Test

The forced swimming test, which assumes that immobility is a measure of behavioral despair, was used to track depressive-like behavior. Two-way ANOVA showed that the stress protocol and the different treatments (saline, perindopril and benazepril) had significant main effect on forced swimming test such as : Immobility time (stress: F(1,30) = 429.8, *p* < 0.0001; different treatments: F(2,30) = 5.162, *p* = 0.0118; stress by treatment: F(2,30) = 7.058, *p* = 0.0031), and Struggling time (stress: F(1,30) = 515.1, *p* < 0.0001; different treatments: F(2,30) = 7.203, *p* = 0.0028; stress by treatment: F(2,30) = 9.607, *p* = 0.0006).

Both perindopril and benazepril effectively reduced CUMS-induced behavioral despair, exhibiting comparable antidepressant-like effects. Specifically, the CUMS group demonstrated a considerably higher immobility time (249.43% increase) and a lower struggling time (78.91% decrease) when compared to the saline control group, confirming the induction of depressive-like behavior.

Conversely, both drug treatments significantly reversed these effects. The perindopril-treated group established a significant decrease in immobility time by 19.19% and an increase in struggling time by 106.91% in contrast to the CUMS group. Similarly, the benazepril-treated group also showed significant improvements, with a 21.21% decrease in immobility time and a 117.26% increase in struggling time.

Importantly, no significant differences were detectedbetween the benazepril- and perindopril-treated groups in terms of immobility or struggling times (Fig. 3C&D), indicating similar efficacy in alleviating behavioral despair.

#### The Splash Test

The splash test, as illustrated in Fig. 3E, was employed to track the self-care and motivational behavior index. Two-way ANOVA showed that the stress protocol and the different treatments (saline, perindopril and benazepril) had significant main effect on the total grooming time (stress: F(1,30) = 58.19, *p* < 0.0001; different treatments: F(2,30) = 11.38, *p* = 0.0002; stress by treatment: F(2,30) = 9.531, *p* = 0.0006).

Our findings reveal that both perindopril and benazepril significantly improved self-care behavior in CUMS-exposed rats, demonstrating comparable, robust effects.Specifically, the CUMS group exhibited a significant decrease in the time spent grooming by 91.80% compared to the saline control group, indicating impaired self-care and motivational deficits.

Conversely, both drug treatments notably reversed this deficit. The perindopril-treated group demonstrated a substantial increase in time spent grooming by 950.00% compared to the CUMS group. Similarly, the benazepril-treated group also showed a significant increase in grooming time by 586.67% relative to the CUMS group. Importantly, there was significant difference in the time spent grooming between the benazepril- and perindopril-treated groups.The benazepril-treated group exhibited a 38.25% decrease in grooming time compared to the perindopril-treated group.

###  Histopathological Examination

#### Hematoxylin and Eosin Stain

##### The Evaluation of the Cortical Tissues

**Fig. 4:**
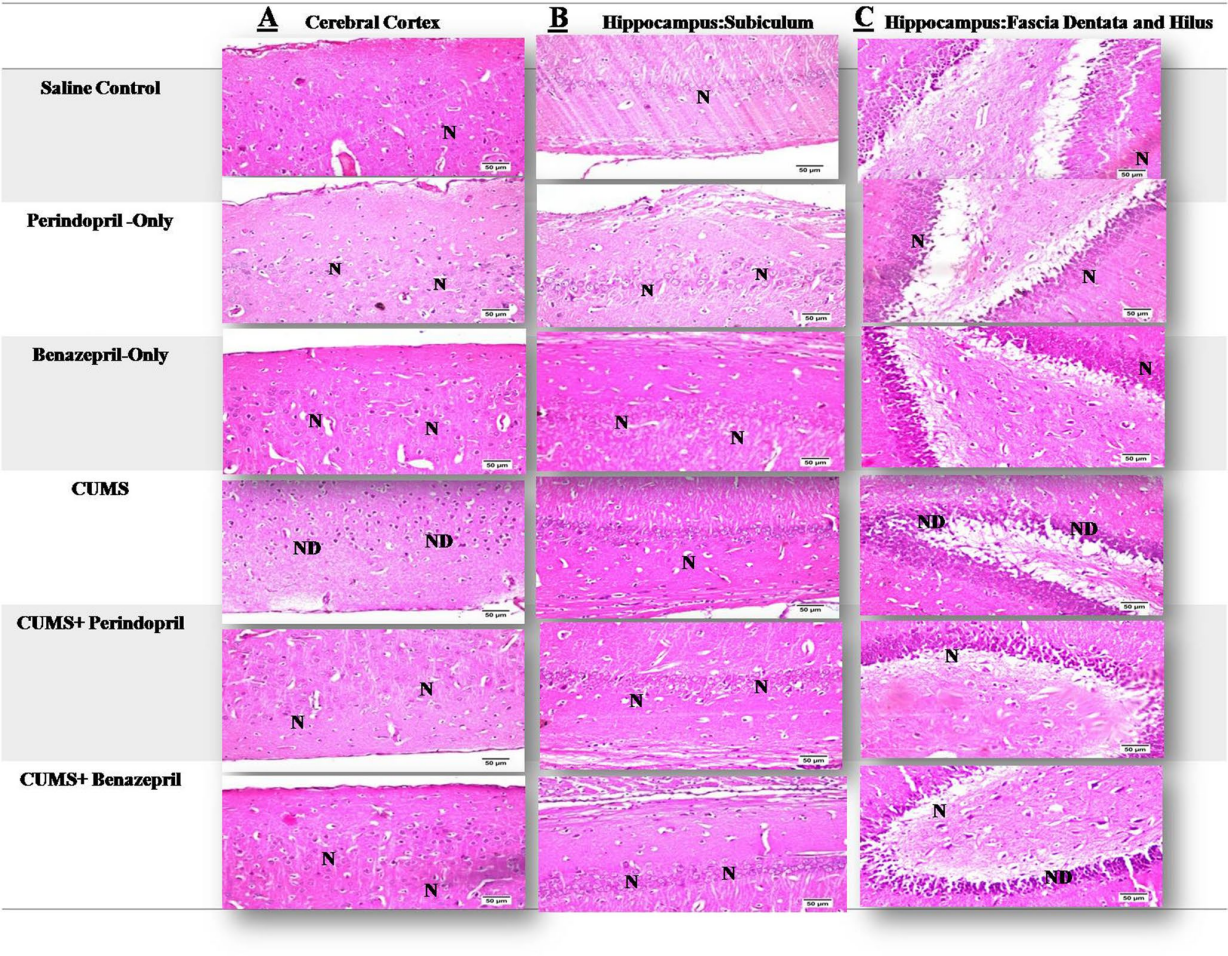
The effects of Perindopril and benazepril on the different brain regions histological characteristics in rats that were exposed to the mild, prolonged, unpredictable stress using Hematoxylin and Eosin stain. Scale bar = 50 μm (x40 magnification) in the cortex, hippocampus/subiculum, and hippocampus/facia dentate and hilus, respectively; The following are the acronyms: N: for normal; ND: for neuron degeneration; and CUM: for Chronic Unpredictable Mild Stress

Our histological examination of cortical tissues (Fig. 4A and Table 2) gave us some compelling evidence: both perindopril and benazepril effectively prevented CUMS-induced neuronal damage, maintaining normal neuronal histology comparable to healthy controls.

**Table 2:** The severity of histopathological changes in various brain regions of various experimental groups

GP Name	Saline control		CUMS		CUMS + Perindopril		CUMS + Benazepril		Perindopril- Only		Benazepril-only	
**Region**	CC	F.HIP	CC	F.HIP	CC	F.HIP	CC	F.HIP	CC	F.HIP	CC	F.HIP
**Nuclar pyknosis & Deg. of neurons**	-	-	+++	++	-	-	-	+	-	-	-	-
**Focal Gliosis**	-	-	-	-	-	-	-	-	-	-	-	-
**Plagues formation**	-	-	-	-	-	-	-	-	-	-	-	-

**+++** Severe (75–100%), **++** Moderate (50–75%), **+** Mild (25–50%), and -Nil (0–25%)

The following are the acronyms: CC: stands for Cerebral cortex, and F.HIP: for Fascia Hippocampus

Specifically, the saline control, perindopril-only, and benazepril-only groups all demonstrated normal histological features of neurons. In stark contrast, the CUMS group exhibited clear signs of neurodegeneration, characterized by degeneration and severe nuclear pyknosis in every neuron. On the other hand, both the benazepril- and perindopril-treated groups displayed normal neuronal histology. This powerfully demonstrates their protective effects against the changes CUMS caused. These findings together suggest that both treatments have a comparable ability to protect the neurons in the cortical region.

##### Evaluation of the Subiculum (hippocampus) Tissues

The subiculum region of the hippocampi of all experimental groups, even the CUMS-exposed animals, showed normal histological features. This finding suggests that the subiculum region was largely unaffected by the CUMS protocol or that the observed CUMS-induced damage was localized to other brain regions(Fig. 4B).

##### Evaluation of the Hilus and Fascia Dentate (hippocampus) Tissues

Histological evaluation of the hilus and fascia dentata regions within the hippocampus (Fig. 4C; Table 2) provided some critical insights into how perindopril and benazepril differed in protecting against CUMS-induced damage; it seemed perindopril offered more complete protection.

As expected, the saline control, perindopril-only, and benazepril-only groups did not exhibit any histological changes. Conversely, sections from the CUMS group displayed substantial nuclear pyknosis and degeneration in several neurons, highlighting significant neuronal harm.

On the other hand, neurons in sections from the perindopril-treated group showed no histological change, indicating complete neuroprotection. The benazepril-treated group, however, displayed only mild neurodegeneration and nuclear pyknosis, suggesting partial, but not complete, protection in these specific hippocampal regions.

#### Nissl Stain

**Fig. 5:**
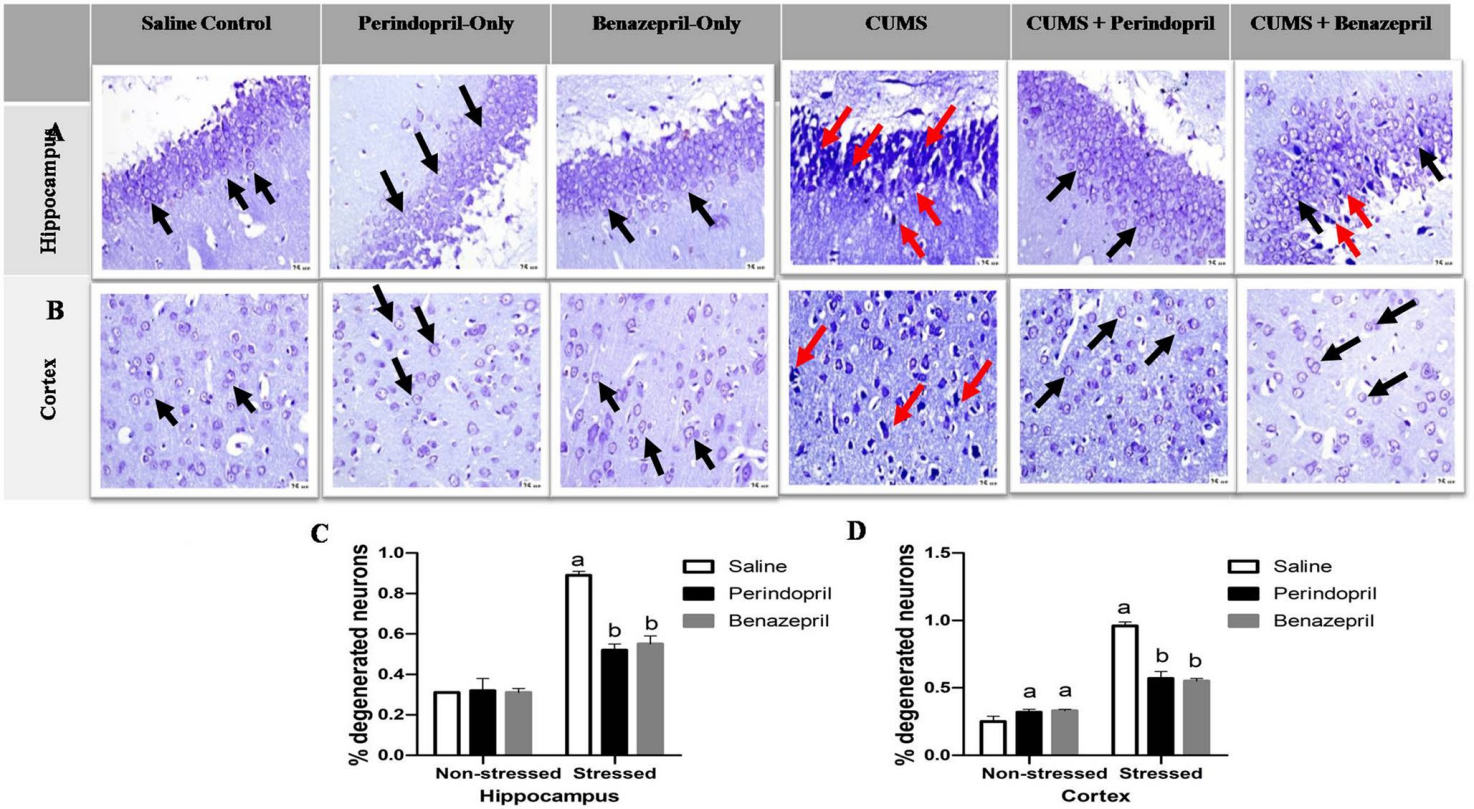
The effects of Perindopril and benazepril on the different brain regions histological characteristics in rats that were exposed to the mild, prolonged, unpredictable stressusing Nissl’s Stain. **A & B **Photomicrographs showing the hippocampus and cortex nissl staining, respectively, Scale bar = 25 μm(x40 magnification). **C & D **The percent of the degenerated neurons to the total neurons by the quantitative image analysis for Nissl staining in the hippocampus and cortex regions, respectively.The means ± standard deviation (*n* = 3) are used to display the data. Bonferroni’s multiple comparison test was used after a two-way ANOVA for statistical analysis. At a significance level of *p* < 0.05, Significant differences from non-stressed saline group (saline control group), Stressed saline group (CUMS group), and Stressed perindopril-treated group (perindopril-treated group)(10 mg/kg) are indicated by labels a, b, and c, respectively. The acronym: CUMS is for for chronic unpredictable mild stress. Black and red arrows indicate intact and pyknotic neurons, respectively

Nissl stain expression in the hippocampus and cortex is depicted in Fig. 5. Two-way ANOVA showed that the stress protocol and different treatments (saline, perindopril and benazepril) had significant main effect on the percentage of degenerated neurons in the hippocampus (stress: F(1,12) = 452.1, *p* < 0.0001; different treatments: F(2,12) = 53.36, *p* < 0.0001; stress by treatment: F(2,12) = 56.84, *p* < 0.0001). Furthermore, two-way ANOVA showed that the stress protocol and different treatments (saline, perindopril and benazepril) had significant main effect on the percentage of degenerated neurons in the cortex (stress: F(1,12) = 708.0, *p* < 0.0001; different treatments: F(2,12) = 53.75, *p* < 0.0001; stress by treatment: F(2,12) = 115.1, *p* < 0.0001).

Statistical analysis revealed that while perindopril offered complete protection against CUMS-induced neuronal damage in both the hippocampus and cerebral cortex, the neuroprotective effect of benazepril was more pronounced in the cortex than in the hippocampus.

Specifically, neurons in both the hippocampal and cerebral cortical regions of the saline control group, as well as the perindopril-only and benazepril-only groups, appeared normal. In stark contrast, the CUMS group displayed a large number of darkly stained, degenerating neurons in both the cerebral cortex and hippocampal regions.

The perindopril-treated group exhibited normal neurons in both the hippocampus and cerebral cortex, indicating a complete reversal of CUMS-induced damage in these areas. Conversely, while the cerebral cortex of the benazepril-treated group appeared normal, its hippocampus still displayed a few dark, degenerating neurons, suggesting partial neuroprotection in this specific region.

On the other hand, there is a significant difference between perindopril only group, benazepril only group and saline control group in cortical percent of degenerated neurons.

### The Effects of Perindopril and Benazepril on Brain monoamine levels in the Rats Exposed To CUMS

**Fig. 6:**
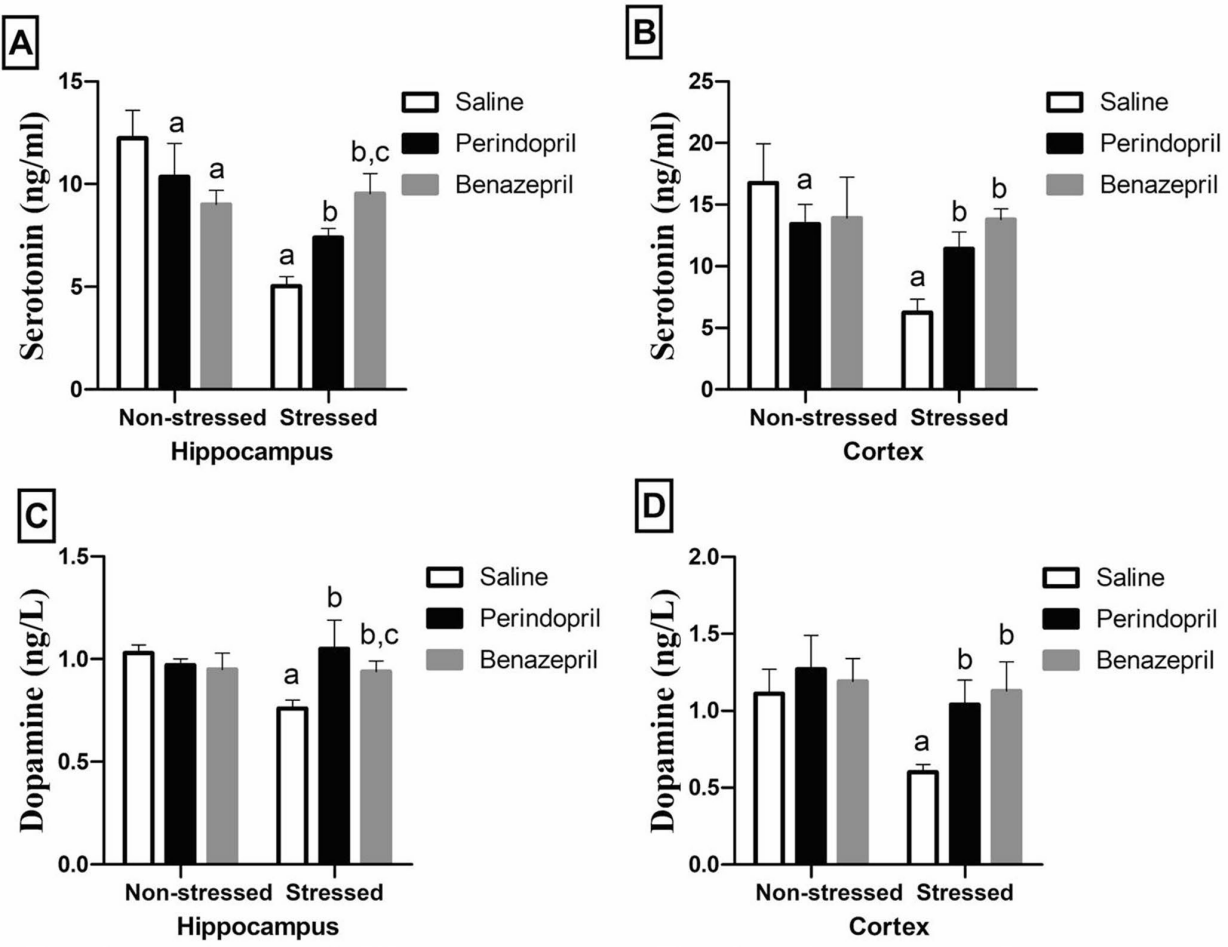
The effects of perindopril and benazepril on the monoamines, serotonin, and dopamine levels in rats’ brain that were exposed to the mild, prolonged, unpredictable stress. **A **& **B **Serotonin expression in the hippocampus and cortex regions, respectively, **C** & **D **Dopamine expression in the hippocampus and cortex regions, respectively. The means ± standard deviation (*n* = 6) are used to display the data. Bonferroni’s multiple comparison test was used after a two-way ANOVA for statistical analysis. At a significance level of *p* < 0.05, Significant differences from non-stressed saline group (saline control group), Stressed saline group (CUMS group), and Stressed perindopril-treated group (perindopril-treated group)(10 mg/kg) are indicated by labels a, b, and c, respectively. The acronyms: CUMS is for chronic unpredictable mild stress

As illustrated in Fig. 6, two-way ANOVA revealed that the stress protocol had significant main effect on hippocampal serotonin level (stress: F(1,30) = 86.89, *p* < 0.0001; stress by treatment: F(2,30) = 42.12, *p* < 0.0001). without any significant effect for treatments, Furthermore, two-way ANOVA showed that the stress protocol and the different treatments (saline, perindopril and benazepril) had significant main effect on cortical serotonin level (stress: F(1,30) = 34.76, *p* < 0.0001; different treatments: F(2,30) = 3.703, *p* = 0.0365; stress by treatment: F(2,30) = 20.12, *p* < 0.0001).On the other hand, it showed that the stress protocol and the different treatments (saline, perindopril and benazepril) had significant main effect on hippocampal dopamine level (stress: F(1,30) = 7.362, *p* = 0.0109; different treatments: F(2,30) = 7.344, *p* = 0.0025; stress by treatment: F(2,30) = 18.24, *p* < 0.0001). Furthermore, it showed the stress protocol and different treatments (saline, perindopril and benazepril) had significant main effect cortical dopamine level (stress: F(1,30) = 23.90, *p* < 0.0001; different treatments: F(2,30) = 13.67, *p* < 0.0001; stress by treatment: F(2,30) = 5.783, *p* = 0.0075.

These results indicate that while both perindopril and benazepril effectively reversed CUMS-induced reductions in serotonin and dopamine, there was a significant difference in the ability of benazepril and perindopril to restore serotonin and dopamine levels, in hippocampal levels.

Specifically, the CUMS group showed significantly lower levels of serotonin and dopamine compared to the saline control group. In the hippocampus, serotonin dropped by 58.87% and dopamine by 26.21%. In the cortex, serotonin was down by 62.75% and dopamine by 45.95%.

Both drug administrations markedly countered these deficits. Perindopril significantly raised serotonin and dopamine levels in the hippocampus by 46.92% and 38.16%, respectively, and in the cortex by 82.85% and 73.33%, respectively, when compared to the CUMS group. Benazepril also significantly increased these neurotransmitters in the hippocampus by 89.46% (serotonin) and 23.68% (dopamine), and in the cortex by 121.31% (serotonin) and 88.33% (dopamine).

A key distinction emerged in the hippocampus: benazepril markedly increased serotoninlevels by 28.96% and decreased dopamine levels by 10.48%, respectively, compared to perindopril-treated CUMS rats. While cortical dopamine levels, as well as cortical serotonin levels, did not significantly differ between the benazepril- and perindopril-treated groups, this specific finding highlights a unique benefit of benazepril in restoring hippocampal serotonin and perindopril in resorting hippocampal dopamine.

both benazepril only group and perindopril only group showed a significant difference in serotonin levels in the hippocampus compared to the saline control group. But in cortical serotonin level the benazepril only group showed a significant difference compared to saline control group.

### The Effects of Perindopril and Benazepril on Brain Inflammatory Markers in the Rats Exposed to CUMS

Two-way ANOVA showed that the stress protocol and the different treatments (saline, perindopril and benazepril) had a significant main effect on hippocampal TNF-α levels(stress: F(1,30) = 91.05, *p* < 0.0001; different treatments: F(2,30) = 10.50, *p* = 0.0003; stress by treatment: F(2,30) = 19.38, *p* < 0.0001). Furthermore, on cortical TNF-α level(stress: F(1,30) = 64.80, *p* < 0.0001; different treatments: F(2,30) = 7.301, *p* = 0.0026; stress by treatment: F(2,30) = 15.77, *p* < 0.0001).

These statistical findings indicate both perindopril and benazepril effectively suppressed CUMS-induced increases in TNF-α expression in the hippocampus and cortex, demonstrating comparable anti-inflammatory effects.

**Fig. 7:**
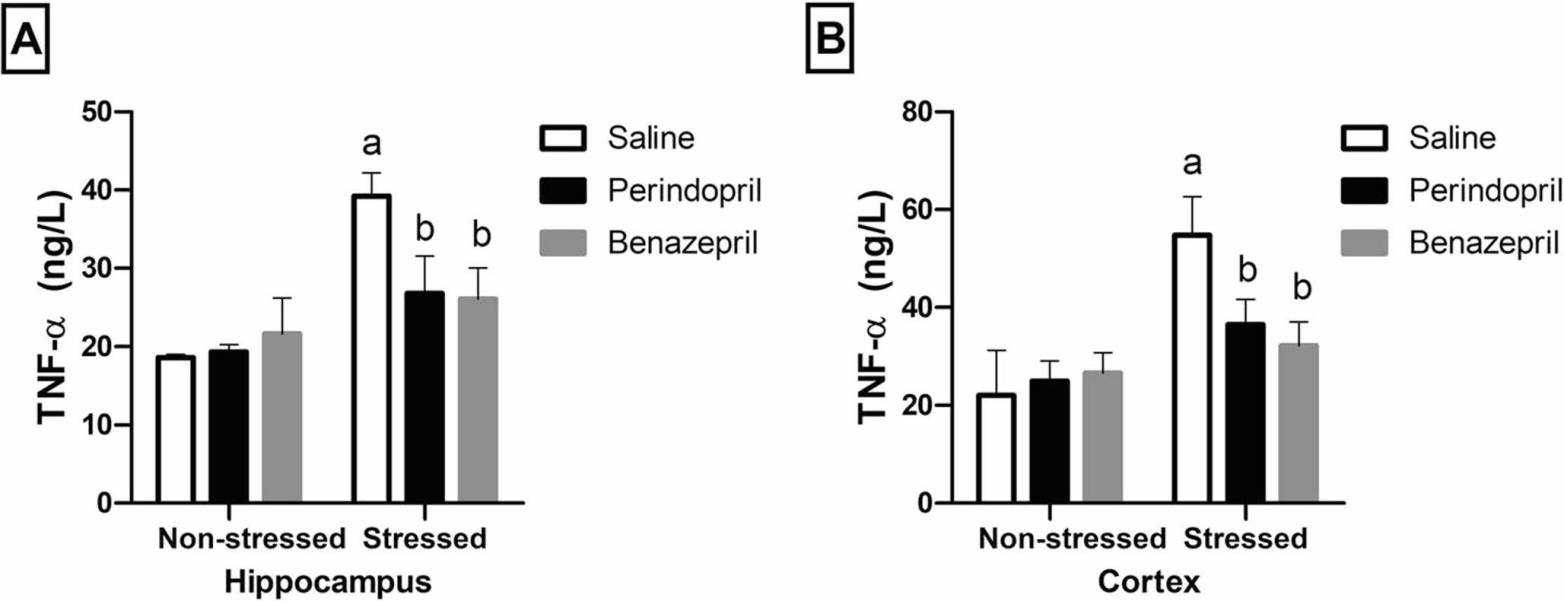
The effects of perindopril and benazepril on the TNF-α inflammatory indicators, in rats that were exposed to the mild, prolonged, unpredictable stress. **A** & **B **TNF-α expression in the hippocampus and cortex regions, respectively. The means ± standard deviation (*n* = 6) are used to display the data. Bonferroni’s multiple comparison test was used after a two-way ANOVA for statistical analysis. At a significance level of *p* < 0.05, Significant differences from non-stressed saline group (saline control group), Stressed saline group (CUMS group), and Stressed perindopril-treated group (perindopril-treated group)(10 mg/kg) are indicated by labels a, b, and c, respectively. The following are the acronyms: TNF-α stands for Tumor necrosis factor alpha, and CUMS for chronic unpredictable mild stress

As demonstrated in Fig. 7, the CUMS group exhibited a significant elevation in hippocampal and cortical TNF-α levels by 110.91% and 148.50%, respectively, compared to the saline control group. Conversely, both drug treatments significantly lowered TNF-α expression. The perindopril-treated group showed reductions in hippocampal and cortical TNF-α by 31.81% and 33.21%, respectively, when compared to the CUMS group. Similarly, the benazepril-treated group also significantly reduced TNF-α expression in the hippocampus by 33.60% and in the cortex by 41.14%.

Importantly, no statistically significant difference in cortical and hippocampal TNF-α levels was detected between the benazepril- and perindopril-treated CUMS groups, suggesting similar efficacy in modulating this pro-inflammatory cytokine.

**Fig. 8:**
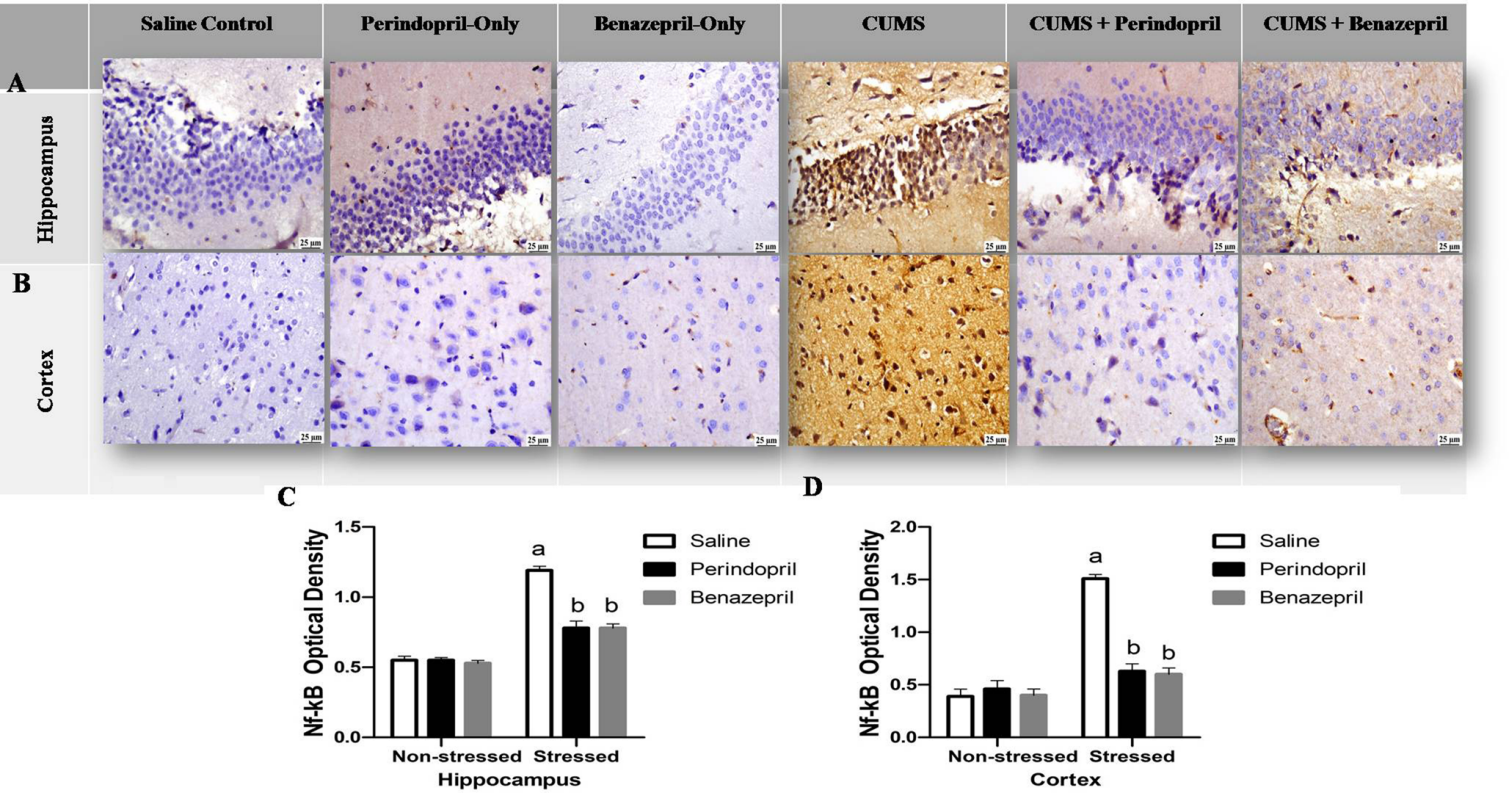
The effects of perindopril and benazepril on the expression of the Immunohisto-chemical inflammatory markers NF-қB in rats that were exposed to the mild, prolonged, unpredictable stress. **A **Photomicrograph, Scale bar = 25 μm(x40 magnification), illustrating NF-қB immunohisto-chemical staining in the hippocampus. **B **Photomicrograph, Scale bar = 25 μm, illustrating NF-қB immunohisto-chemical staining in the Cortex. **C **&** D **Quantitative image analysis for NF-қB expression as optical densities (OD) in the hippocampus and cortex regions, respectively.The means ± standard deviation (*n* = 3) are used to display the data. Bonferroni’s multiple comparison test was used after a two-way ANOVA for statistical analysis. At a significance level of *p* < 0.05, Significant differences from non-stressed saline group (saline control group), Stressed saline group (CUMS group), and Stressed perindopril-treated group (perindopril-treated group)(10 mg/kg) are indicated by labels **a**,** b**,** and c**, respectively. The following are the acronyms: NF-қB: stands for nuclear factor kappa- B, and CUMS for chronic unpredictable mild stress

Furthermore, the immunohistochemical evaluation of NF-κB expression in the hippocampus and cortex, as shown in Fig. 8A & B, provided additional insights. Two -way ANOVA showed that the stress protocol and the different treatments (saline, perindopril and benazepril) had significant main effect on hippocampal NF-κB expression (stress: F(1,12) = 627.2, *p* < 0.0001; different treatments: F(2,12) = 88.35, *p* < 0.0001; stress by treatment: F(2,12) = 80.15, *p* < 0.0001), and cortical NF-κB expression (stress: F(1,12) = 266.4, *p* < 0.0001; different treatments: F(2,12) = 88.45, *p* < 0.0001; stress by treatment: F(2,12) = 105.0, *p* < 0.0001).

These statistical findings indicate both perindopril and benazepril effectively suppressed CUMS-induced increases in NF-κB expression in the hippocampus and cortex, demonstrating comparable anti-inflammatory effects.

Notably, NF-κB expression in the CUMS group was notably higher than that of the saline control group, increasing by 116.36% in the hippocampus and 287.18% in the cortex. Both perindopril and benazepril administrations led to significant reductions in NF-κB expression compared to the CUMS group. Specifically, the perindopril-treated group demonstrated a 34.45% decrease in NF-κB expression in the hippocampal region and a 58.28% decrease in the cerebral cortex. Likewise, the benazepril-treated group also showed a substantial 34.45% decrease in NF-κB expression in the hippocampal region and a 60.26% decrease in the cerebral cortex.

Crucially, the benazepril-treated group showed no significantly lower level of NF-κB positive staining when compared to the perindopril-treated group, reinforcing their comparable ability to mitigate this key inflammatory mediator. No significant difference was observed between drugs-only groups (perindopril-only or benazepril-only) in all examined brain regions. Quantitative image analysis of NF-κB expression as optical densities (OD) in the hippocampus and cortex is further exhibited in Fig. 8C & D.

### 3.5 The Effects of Perindopril and Benazepril on Apoptosis indicator, caspase-3, in the Rats’ Brains Exposed To CUMS

**Fig. 9:**
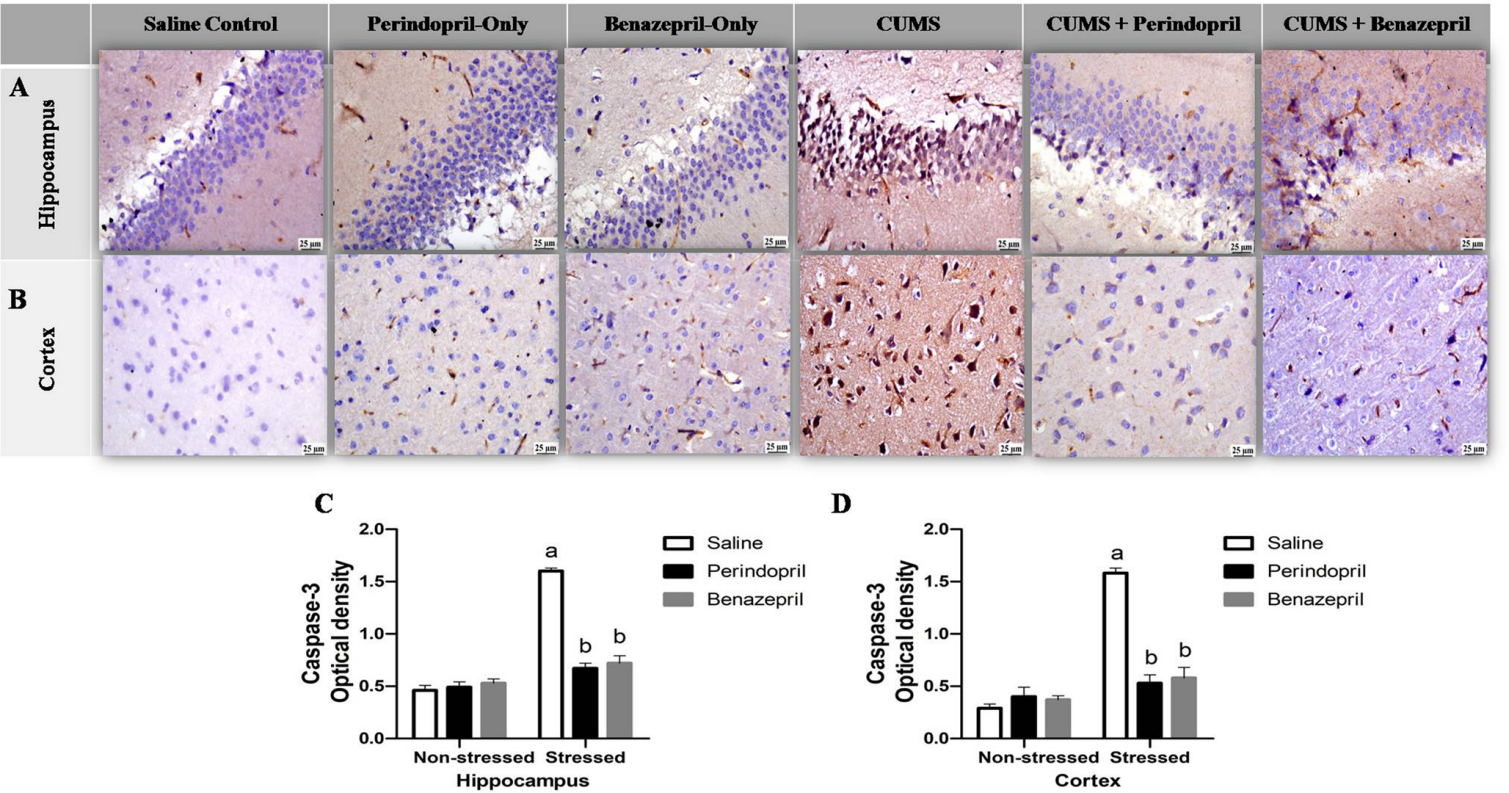
The effects of perindopril and benazepril on the expression of the immunohisto-chemical apoptosis marker as caspase-3 expression in rats that were exposed to the mild, prolonged, unpredictable stress. **A **photomicrograph showing caspase-3 immunohistochemical expression in the hippocampus, with a scale bar of 25 μm and x40 magnification. **B **Photomicrograph showing caspase-3 immunohistochemical expression in the cortex, scale bar = 25 μm and x40 magnification. **C **& **D** Quantitative image analysis of the Hippocampaland Cortical optical densities (OD) for Caspase-3 expression, respectively.The means ± standard deviation (*n* = 3) are used to display the data. Bonferroni’s multiple comparison test was used after a two-way ANOVA for statistical analysis. At a significance level of *p* < 0.05 Significant differences from non-stressed saline group (saline control group), Stressed saline group (CUMS group), and Stressed perindopril-treated group (perindopril-treated group)(10 mg/kg) are indicated by labels a, b, and c, respectively. The following is the acronyms: CUMS stands for chronic unpredictable mild stress

The immunohistochemical evaluation of Caspase-3 expression in the hippocampus and cortex is depicted in Fig. 9A & B. Two -way ANOVA showed that the stress protocol and the different treatments (saline, perindopril and benazepril) had significant main effect on hippocampal caspase-3 expression (stress: F(1,12) = 459.1, *p* < 0.0001; different treatments: F(2,12) = 148.4, *p* < 0.0001; stress by treatment: F(2,12) = 183.6, *p* < 0.0001), and cortical caspase-3 expression (stress: F(1,12) = 263.9, *p* < 0.0001; different treatments: F(2,12) = 85.95, *p* < 0.0001; stress by treatment: F(2,12) = 125.1, *p* < 0.0001).

These statistical findings indicate that both perindopril and benazepril effectively reduced CUMS-induced Caspase-3 expression, demonstrating comparable anti-apoptotic effects, though with notable differences in the extent of suppression.

Specifically, the saline control group, perindopril-only group, and benazepril-only group all displayed extremely low to negative Caspase-3 expression in both the hippocampus and cerebral cortex. In contrast, the CUMS group showed significantly higher levels of Caspase-3 expression in the cerebral cortex and hippocampal regions, by 444.83% and 247.83%, respectively, compared to the saline control group, indicating increased apoptosis.

Both treated groups significantly lowered Caspase-3 expression compared to the CUMS group. The perindopril-treated group exhibited limited Caspase-3 expression in the cerebral cortex and hippocampal regions, with reductions of 58.13% and 66.46%, respectively. The benazepril-treated group also showed a decrease in Caspase-3 expression, displaying mild positive staining in the cerebral cortex and hippocampal regions by 63.29% and 55.00%, respectively.

Importantly, there was no discernible change in Caspase-3 positive staining in the benazepril-treated group when compared to the perindopril-treated group, suggesting similar anti-apoptotic efficacy despite the numerical differences in reduction percentages. Furthermore, the quantitative image analysis of Caspase-3 expression as optical densities (OD) in hippocampus and cortex areas is shown in Fig. 9C & D.

### The Effects of Perindopril and Benazepril on Brain RAS Levels in the Rats Exposed to CUMS

Two-way ANOVA showed that the stress protocol and the different treatments (saline, perindopril and benazepril) had significant main effect on hippocampal Ag II expression (stress: F(1,12) = 215.8, *p* < 0.0001; different treatments: F(2,12) = 26.65, *p* < 0.0001; stress by treatment: F(2,12) = 32.37, *p* < 0.0001), and cortical Ag II expression (stress: F(1,12) = 347.7, *p* < 0.0001; different treatments: F(2,12) = 25.45, *p* < 0.0001; stress by treatment: F(2,12) = 26.22, *p* < 0.0001). However, Two ANOVA showed that the stress protocol and the different treatments (saline, perindopril and benazepril) had significant main effect on Hippocampal ATR2 expression (stress: F(1,12) = 611.2, *p* < 0.0001; different treatments: F(2,12) = 34.30, *p* < 0.0001; stress by treatment: F(2,12) = 49.38, *p* < 0.0001), and cortical ATR2 expression (stress: F(1,12) = 190.5, *p* < 0.0001; different treatments: F(2,12) = 13.48, *p* = 0.0009; stress by treatment: F(2,12) = 19.37, *p* = 0.0002).

Our investigation into the brain’s Renin-Angiotensin System (RAS) components revealed that while both perindopril and benazepril effectively suppressed CUMS-induced increases in Ag II and ATR2 receptor levels, benazepril demonstrated a superior ability to reduce these markers in both the hippocampus and cortex.

**Fig. 10:**
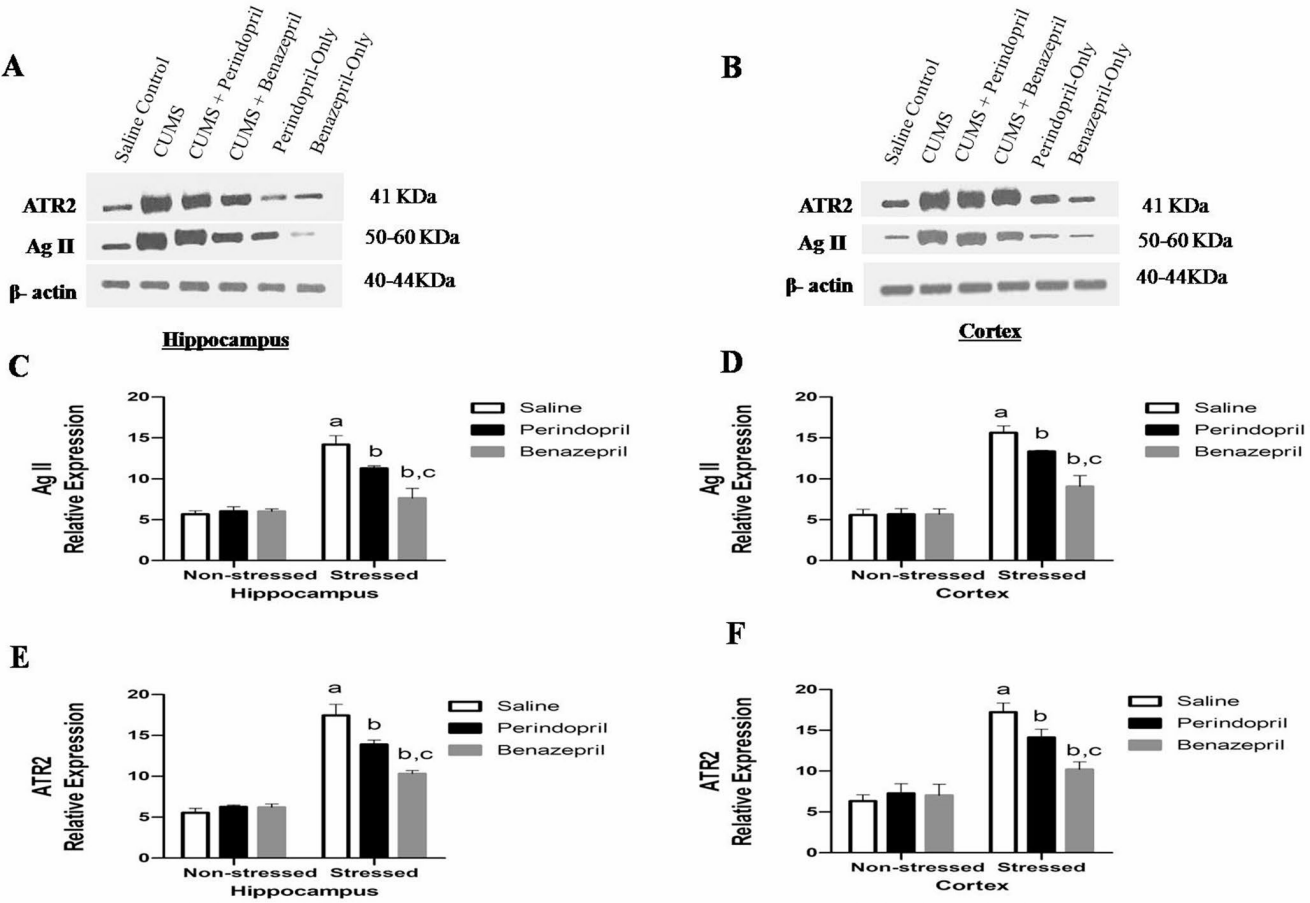
The effects of perindopril and benazepril on the AgII and ATR2 receptor brain contents in rats that were exposed to the mild, prolonged, unpredictable stress. **A **Ag II protein expression level and ATR2 protein expression level for hippocampal samples in different experimental groups compare to β- actin expression level for hippocampal samples, **B **Ag II protein expression level and ATR2 protein expression level for cortical samples in all groups as compare to expression level of β actin proein expression level for cortical samples. **C **&** E **The effect of perindopril and benazepril on the Hippocampal AgII and ATR2 respectively, in the CUMS-exposed rats. **D **&** F **The effect of perindopril and benazepril on the cortical Ag II and ATR2, respectively, in the CUMS-exposedrats. The means ± standard deviation (*n* = 3) are used to display the data. Bonferroni’s multiple comparison test was used after a two-way ANOVA for statistical analysis. At a significance level of *p* < 0.05, Significant differences from non-stressed saline group (saline control group), Stressed saline group (CUMS group), and Stressed perindopril-treated group (perindopril-treated group)(10 mg/kg) are indicated by labels a, b, and c, respectively. The following are the acronyms: CUMS stands for chronic unpredictable mild stress, Ag II: for Angiotensin II, and ATR2: for Angiotensin type 2 receptor

As demonstrated in Fig. 10, the CUMS group exhibited a significant elevation in Ag II and ATR2 receptor levels compared to the saline control group. Specifically, hippocampal levels increased by 149.47% (Ag II) and 214.80% (ATR2), while cortical levels rose by 178.93% (Ag II) and 172.20% (ATR2).

Conversely, both drug treatments significantly countered these increases. Perindopril administration considerably lowered brain Ag II and ATR2 receptor levels in the hippocampus by 20.25% and 20.30%, respectively, and in the cortex by 14.66% and 17.99%, respectively, when compared to the CUMS group. Benazepril administration also significantly decreased Ag II and ATR2 receptor levels, reducing them in the hippocampus by 46.08% and 40.77%, respectively, and in the cortex by 41.87% and 40.74%, respectively, relative to the CUMS group.

Notably, a key distinction emerged when comparing the two treatments: benazepril administration significantly decreased Ag II and ATR2 receptor levels in the hippocampus by 32.39% and 25.68%, respectively, and in the cortex by 31.88% and 27.74%, respectively, compared to the perindopril-treated groups. This really highlights benazepril’s superior ability to modulate these central RAS components when dealing with the effects of CUMS.

## Discussion

Major depressive disorder (MDD) is a complex condition, stemming from a blend of biological, psychological, environmental, and genetic factors. These factors encompass physiological mechanisms, such as inflammatory processes and monoamine pathways, psychological elements like cognitive patterns, contextual influences like poverty, recent adverse life events, and early-life adversity, and genetic predispositions (Marx et al. 2023). Approximately 20% of the individuals worldwide are affected by depression, a severe and recurrent emotional disorder (Linghu et al. 2020). However, there are two substantial challenges in the treatment of MDD; the response rate to pharmaceutical interventions is only between 40% and 70%, and the therapeutic effects often require a considerable amount of time to become apparent (Y. Luo et al. 2020a, b). The need for more research into antidepressant therapies is a public health concern, and these issues need to be addressed (Gong 2022). This study, by comparing the effects of perindopril and benazeprilon depression in the CUMS rat model, provides novel insights into how different ACE inhibitors might treat this complex disorder. It particularly sheds light on their varied pathways, considering their distinct abilities to access the brain.

Our findings supported the well-established efficacy of the CUMS model in inducing depressive-like behaviors in rats. In the Open Field Test (OFT), as shown in Fig. 2, we noted specific symptoms, including longer reaction times to ambulation as well as a reduction in grooming and rearing activities.These findings are corroborated by further supporting evidence from the forced swimming test (FST), as shown in Fig. 3C & D. Consistent with earlier researches, the FST showed that rats exposed to CUMS had increased immobility time and decreased struggle time.(Abelaira et al. 2013; Hassan et al. 2020; Nazeem et al. 2021). The OFT serves as a valuable method to evaluate locomotor activity, while the FST is a reliable model for assessing the state of despair and depressive behavior in rats. Moreover, the CUMS model provoked broader behavioral deficits, including diminished cognitive performance as measured through the Y-Maze test, and reduced grooming time in the splash test, which represents an index for self-care and motivational behavior, as shown in Fig. 3A, B & E. Taken together, these outcomes are highly consistent with previous research demonstrating the behavioral and neurobiological alterations seen in CUMS-induced models of depression (Abelaira et al. 2013; Hu et al. 2017; Overstreet 2012; Yan et al. 2020).

Our examination of tissue samples showed clear evidence that CUMS caused harm to important brain regions, notably the cortex and specific hippocampal areas, including the fascia dentata and hilus. The presence of nuclear pyknosis and neuronal degeneration in these regions aligns consistently with earlier studies (Hassan et al. 2020; Khan et al. 2018; Liu et al. 2019a, b). This damage to brain structures is linked to the emotional regulation and cognitive impairments commonly observed in individuals with depression (Lu et al. 2017).

To identify the best ACE inhibitor that might reverse these behavioral and histological defects, benazepril and perindopril were administered to the depressed rats in a screening investigation. The specific dosage (10 mg/kg, p.o.) and administration details were thoroughly outlined in the Methods section.

Comparing Benazepril and Perindopril directly was an important part of our research. Interestingly, the CUMS-induced behavior changes were markedly attenuated by the co-treatment with either perindopril or benazepril, with no significant differences observed between them in the Open Field Test (OFT), and Forced Swimming Test (FST), as shown in Figs. 2 and 3. For most behavioral parameters assessed, both drugs exhibited remarkably comparable efficacy, including ambulation, rearing, grooming, and latency in the OFT; immobility and struggling time in the FST; and spontaneous alternation percentage in the Y-maze. This also suggests a similar general therapeutic impact on core depressive-like symptoms.However, notable distinctions emerged in specific parameters, hinting at complex differential effects. In the Y-maze test, perindopril significantly elevated the Total Arm Entries (TAE) compared to benazepril, which implies that a more favorable effect on general exploratory activity for perindopril, despite comparable improvements in spatial alternation between the two drugs.In the splash test, while both drugs significantly improved self-care, the benazepril-treated group showed a significant decrease in grooming time compared to the perindopril-treated group, indicating perindopril led to a more pronounced restoration of motivational behavior. Besides, both perindopril and benazepril alleviated the histopathological aberrations provoked by CUMS exposure. Notably, the benazepril administration showed mild histological alteration in hippocampal neurons, whereas perindopril administration resulted in a normal histopathological structure in these same neurons, as shown in Figs. 4 and 5.

Our study found that CUMS rats had lower levels of serotonin and dopamine, as shown in Fig. 6. This is a well-known finding in depression research, and our results are consistent with previous studies (Feenstra et al. 1996; Fernandes and Gupta 2019; Hassan et al. 2020; Liu et al. 2006; Lu et al. 2019). This reduction in neurotransmitters is consistent with the notion that serotonin dysregulation plays a significant role in depression(Mohammad-Zadeh et al. 2008). Both perindopril and benazepril increased these neurotransmitter levels in cortex and hippocampus tissues. However, a significant difference was observed between them in either hippocampal serotonin or dopamine levels. This specific finding highlights a unique benefit of benazepril in restoring hippocampal serotonin, contributing to its strong behavioral efficacy. On other hand, perindopril demonstrated a more potent effect on resorting hippocampal dopamine.

Additionally, we found that rats subjected to CUMS exhibited signs of chronic inflammatory reactions. In particular, as shown in Fig. 7, we found elevated levels of TNF-α in the hippocampus and cortex, which is consistent with research that links inflammation to depression (Bajpai et al. 2014; Ma et al. 2016).Notably, both Perindopril and benazepril treatment effectively reduced these elevated TNF-α levels, highlighting their potential anti-inflammatory effects with no significant difference observed between them, suggesting similar efficacy in modulating this pro-inflammatory cytokine.This is particularly relevant given that numerous TNF-α antagonists have been applied to block TNF-α-augmented activity in depressed patients (Meroni et al. 2015).

On the other hand, the CUMS group showed an increase in NF-κB tissue levels in both the hippocampus and cortex. These findings are consistent with earlier research on rats’ depression brought on by CUMS (Hassan et al. 2020; Jiang et al. 2020; Zhao et al. 2023). As Shown in Fig. 8, this effect was attenuated by both perindopril and benazepril treatment; however, a clear distinction emerged where perindopril caused significantly more pronounced reductions in NF-κB expression compared to benazepril. This suggests a differential impact on this crucial inflammatory pathway, indicating perindopril’s potentially stronger anti-inflammatory action through this specific mechanism.

In the present study, the CUMS protocol triggered neuronal apoptosis in depressed rats, as proven by an increase in caspase-3 tissue levels, which agrees with previous studies (Hassan et al. 2020; Wu et al. 2023; Zhao et al. 2023). On the other hand, as shown in Fig. 9,by reversing the CUMS-induced caspase-3 increase, both perindopril and benazepril demonstrated an anti-apoptotic impact, while prior research has shown that benazepril and perindopril have anti-inflammatory and antioxidant properties in many animal models (Bryniarski et al. 2022; Gong 2022; Zhan et al. 2021), According to our findings, both effectively reduced the expression of Caspase-3 produced by CUMS, demonstrating comparable anti-apoptotic effects albeit at somewhat varying degrees of suppression. In particular, a significant difference was observed in favor of perindopril in reducing Caspase-3 levels, indicating that it may have a more potent anti-apoptotic effect in this context.

As shown in Fig. 10, the CUMS model resulted in elevated levels of Ang II and ATR2 expression in both the cortex and hippocampus, aligning with earlier research findings (Liu et al. 2019a, b; Yu et al. 2019).The current research observed that treatment with perindopril or benazepril effectively reversed these alterations, significantly decreasing Ang II and ATR2 receptor expression in cortex and hippocampus tissues. A notable difference observed between the two treatment groups: benazepril showed more promising effect on Ag II and ATR2 receptor expression than perindopril, which promotes a stronger neuroprotective effect. This suggests that ACE inhibitors may modulate RAAS-related inflammation and neurotoxicity. The role of Ang II in neuroinflammation and apoptosis, through mechanisms involving NF-κB activation and caspase-3 elevation, was evident in our study. Both ACE inhibitors mitigated these effects, further supporting their neuroprotective potential.

Our findings regarding Ang II and ATR2 expression in our CUMS model strongly support the notion that stress-induced brain damage and depressive behaviors are influenced by an overactive RAAS system. Benazepril and perindopril both had the ability to lower these elevated levels, which was encouraging and strongly suggests that they function by adjusting this RAAS system.Furthermore, considering benazepril’s primarily non-central/peripheral action, our discovery that it demonstrated a more marked decrease in Ag II and ATR2 expression in comparison to perindopril provides a novel mechanistic explanation for its strong behavioral and neuroprotective effects. The way that they impact these important RAAS components varies, suggesting that even ACE inhibitors with low BBB penetration can have a major impact on central RAAS activity. This underscores the intricate relationship between peripheral and central systems in the pathophysiology of depression.

The Ang II functions in the brain are intricate and varied. Although the effects of Ang II through the AT1 receptor are mainly damaging, the compensatory impact of AT2 receptor activation underscores the potential therapeutic benefits of targeting the RAAS in neurological conditions, as indicated by (Biancardi and Stern 2016; Sabuhi et al. 2011). Research has shown associations between Ang II and conditions such as depression, anxiety, HPA axis hyperactivity, and stress (Tashev and Ivanova 2018). The ATR2 has emerged as a potentially crucial factor in tissue repair and neuroregeneration, with increased ATR2 expression observed in various pathological states like nerve crush injuries and inflammation (Danigo et al. 2021). Although its precise role in the peripheral nervous system remains largely unexplored, its involvement in CNS recovery is suggested by heightened ATR2 levels post-brain injuries (Viswanathan and Saavedra 1992). In some pathological condition, such as stroke, ATR2 is elevated, which suggests that it might be engaged in an endogenous response to injury (Li et al. 2005). However, it is well- established that using particular agonists to activate ATR2 following certain neurodegenerative diseases causes nerve tissue to differentiate and regenerate (Reinecke et al. 2003). It has been linked that over-activation of brain RAS is associated with cognitive impairment and the development of neurodegenerative diseases (Wu et al. 2022).

Beyond its suggested involvement in tissue restoration, ATR2 is intricately associated with the adjustment of the extracellular microenvironment in neurons. This role is exemplified by its control of serine protease inhibitors in Schwann cells (Bleuel et al. 1995). Nonetheless, the relationship between ATR2 and cellular functions is intricate. Although elevated ATR2 levels often coincide with neuroprotective reactions, excessive expression of ATR2 by itself can paradoxically trigger apoptosis independently of Ang II binding (Miura and Karnik 2000). This apoptotic pathway involves the activation of p38 MAPK and caspase 3.

Adding thorough entanglement to this process, ATR2 expression is also significantly influenced by inflammatory factors. Specifically, IL-1 and interferon-gamma can increase ATR2 expression through NF-kB and JAK/STAT pathways (Kaschina and Unger 2003; Ruiz-Ortega et al. 2000). This indicates a possible involvement of ATR2 in both the initiation and resolution of inflammatory responses. In summary, ATR2 plays a dual role in neurobiology, exhibiting both protective and potentially detrimental effects depending on the context and level of activation. A comprehensive understanding of the mechanisms governing these contradicting actions is crucial for the development of targeted therapeutic interventions, especially when considering RAAS modulation in neuropsychiatric conditions.

The present research emphasizes the potential of ACEIs as a promising treatment option for depression, highlighting their significant influence on the Renin-Angiotensin System (RAAS), neuroinflammation, and neuroprotection. While our initial findings are highly encouraging, further additional studies are needed to delve into the specific mechanisms behind the efficacy of ACEIs in treating depression, as well as their potential long-term impacts. Filling these knowledge gaps, particularly regarding the differential actions of individual ACE inhibitors and their precise molecular targets, could significantly advance the development of more efficient antidepressant treatments and ultimately enhance patient outcomes.

## Conclusion

In summary, our study offers novel and significant insights into the therapeutic potential of angiotensin-converting enzyme inhibitors (ACEIs), perindopril and benazepril in mitigating oxidative stress, inflammation, and apoptosis, as well as inhibiting the renin-angiotensin-aldosterone system (RAAS) in chronic unpredictable mild stress (CUMS) model in rats. This study targets in-depthinvestigation into the antidepressant potential of benazepril, a non-central acting ACE inhibitor, comparing it directly to perindopril, a well-known central-acting ACE inhibitor. Our precise comparative analysis revealed remarkable similarities in their overall efficacy across multiple behavioral tests (including locomotor activity, exploration, memory, despair, and self-care), as both successfully counteracted the neuroinflammatory responses and neuronal damage induced by chronic stress in our rat model. This comprehensive assessment, however, also underscored intriguing subtle differences, as each ACEI demonstrated unique advantages and superior results in certain aspects compared to the other.Specifically, perindopril exhibited superior performance in the splash test (total grooming time) and Y-maze (total arm entries), along with more comprehensive neuroprotection in certain hippocampal regions. Conversely, benazepril showed more complete variations in other aspects of exploratory activity and self-care.

**Fig. 11:**
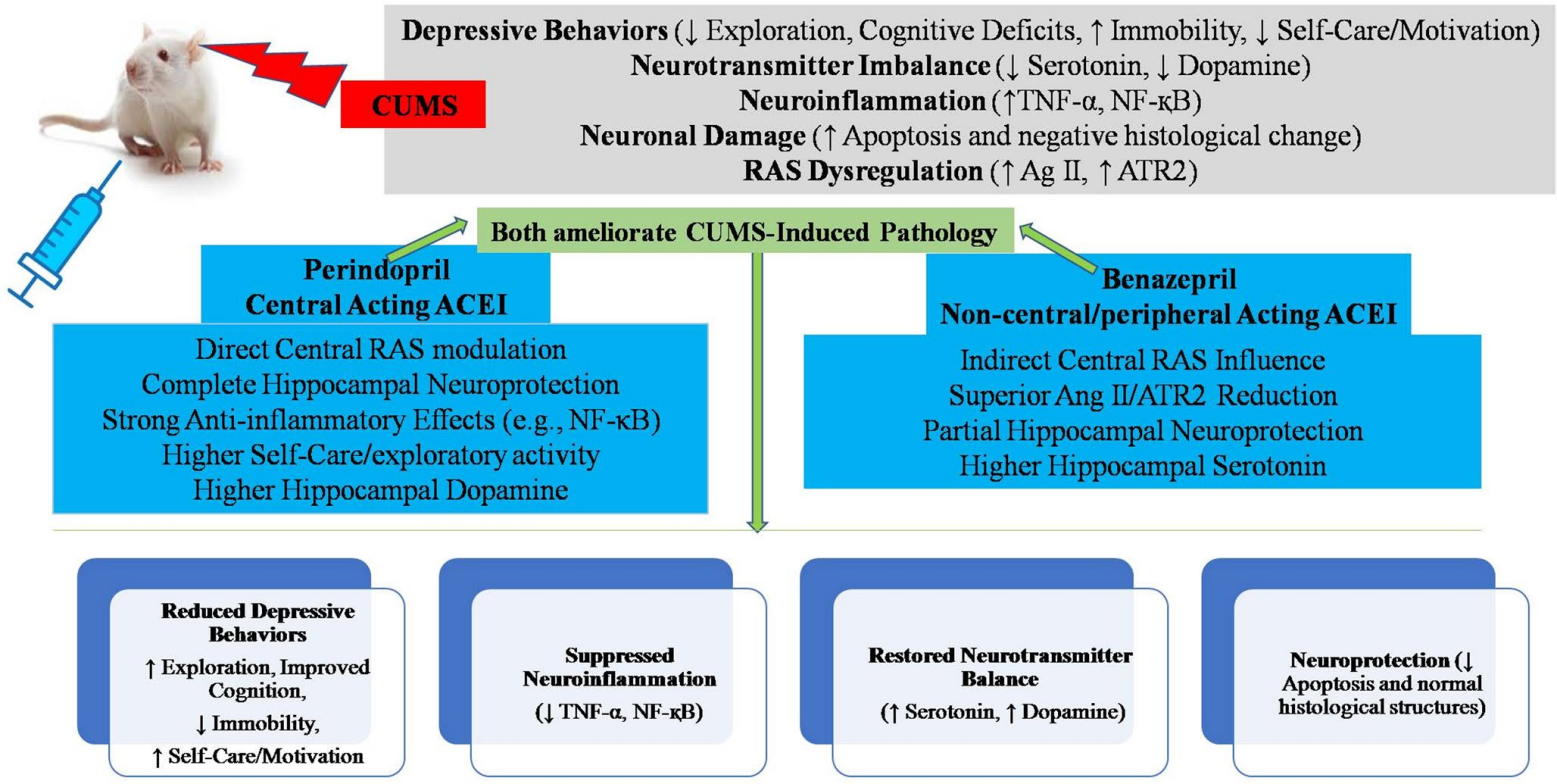
Comparative Neurobiological and Behavioral Effects of Perindopril and benazepril in rats that were exposed to the mild, prolonged, unpredictable stress. The following are the acronyms: CUMS stands for chronic unpredictable mild stress, TNF-α stands for Tumor necrosis factor alpha, NF-қB: stands for nuclear factor kappa- B, Ag II: for Angiotensin II, and ATR2: for Angiotensin type 2 receptor

Mechanistically, the current research thoroughly explored how these drugs exert their effects, from rebalancing neurotransmitter levels to suppressing neuroinflammation (TNF-α and NF-κB) and reducing apoptosis (Caspase-3). importantly, the present results showed that benazepril, despite its limited blood-brain barrier penetration, demonstrated a more marked reduction in Ag II and ATR2 levels than perindopril, offering a novel mechanistic explanation for its strong efficacy. The strong behavioral efficacy of benazepril may be explained by its superior modulation of Ag II/ATR2 signaling and more effective restoration of hippocampal serotonin levels. Even though it has direct anti-inflammatory properties, this offers a different means of explaining its potent behavioral effects, and NF-kB effects may have been less severe than those of perindopril. In addition to, stronger anti-inflammatory effects of perindopril by more significantly suppressing NF-κB, it had a superioreffect in restoring hippocampal dopamine levels.The findings imply that ACE inhibitors with a primarily non-central/peripheral action may have a significant effect on central RAS activity, potentially via indirect pathways.A visual overview of these key mechanistic insights and their corresponding results is provided on Fig. 11. However, it remains uncertain whether all ACE inhibitors possess similar antidepressant effects, and while this study establishes the efficacy of perindopril and benazepril, their different mechanisms—particularlycentral versus non-central action—highlight the urgent need for further research to clarify the relationship between BBB permeability and neuroprotection. These insights are crucial for developing targeted antidepressant therapies and underscore the importance of continued exploration into the full range of ACEIs.

## Study Limitations

Although our research provides valuable insights, it is crucial to recognize its limitations. First, even though the CUMS rat model was sufficiently used in the current study, further research models might be requested to clinically represent the complexity of MDD in humans. Caution is thus necessary when extrapolating these results directly to clinical practice. Second, this research was limited to male rats, thereby ignoring possible sex-specific variations in RAAS and stress reactions that might affect the effectiveness of the medication. Finally, though a number of neurobiological pathways have been explored, more thorough research is necessary to determine the exact mechanisms by which benazepril produces its central effects due to its limited BBB penetration. These conclusions would be strengthened by direct evidence from investigations into alterations in BBB integrity or in vitro cellular assays. Hence, these study limitations draw attention to important research areas that require further exploration.

## Supplementary Information

Below is the link to the electronic supplementary material.

Supplementary Material 1 (PDF 625 KB)

## Data Availability

Research data will be available upon reasonable request.
