# Supplementary material for: Comparative Study on the Neuroprotective Effects of Perindopril and Benazepril in Experimentally-induced Chronic Mild Stress in Rats
Source: J Neuroimmune Pharmacol. 2025 Oct 9;20(1):84. doi: 10.1007/s11481-025-10244-z (PMC12511162; doi:10.1007/s11481-025-10244-z)

Frist Beta Actin
3 image for cortex

Beta Actin 1 (Cortex)

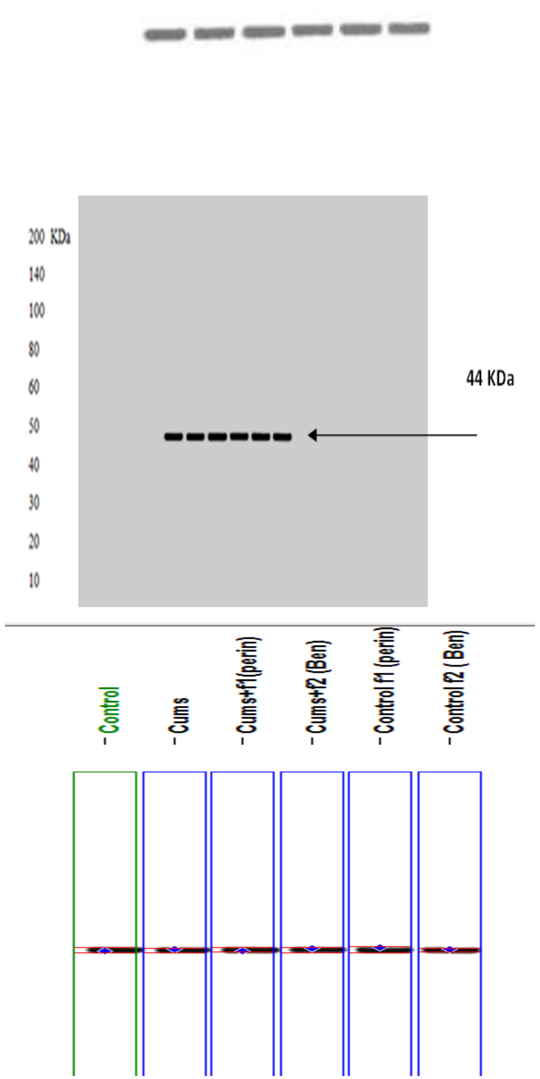

Beta Actin 2 (Cortex)

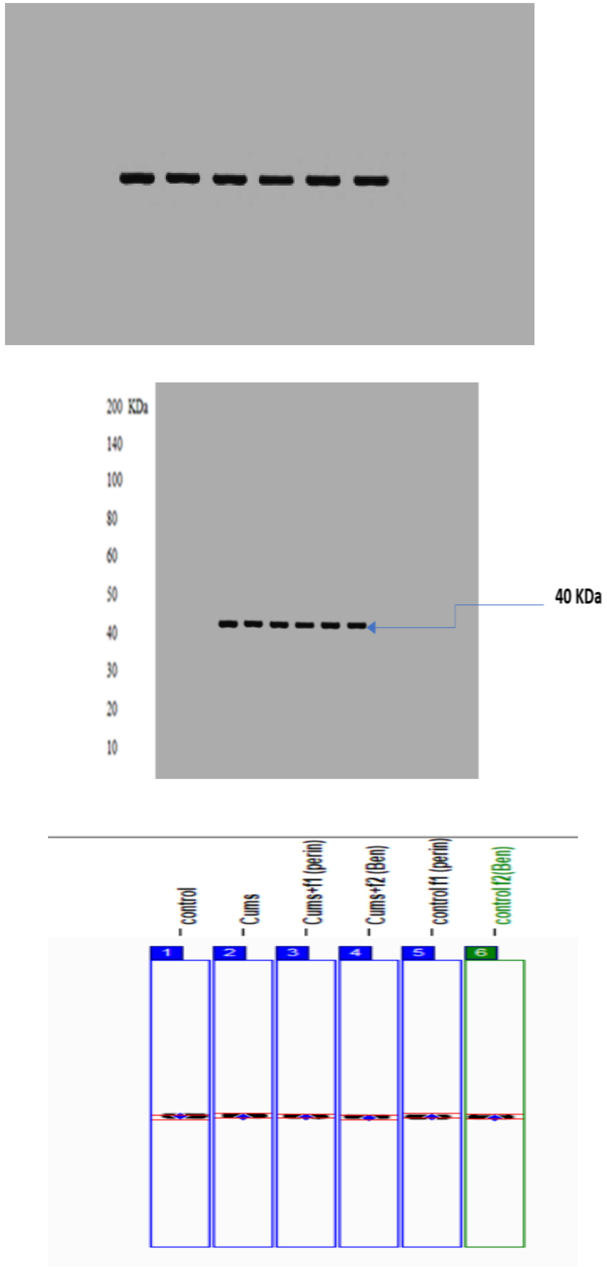

Beta Actin 3 (Cortex)

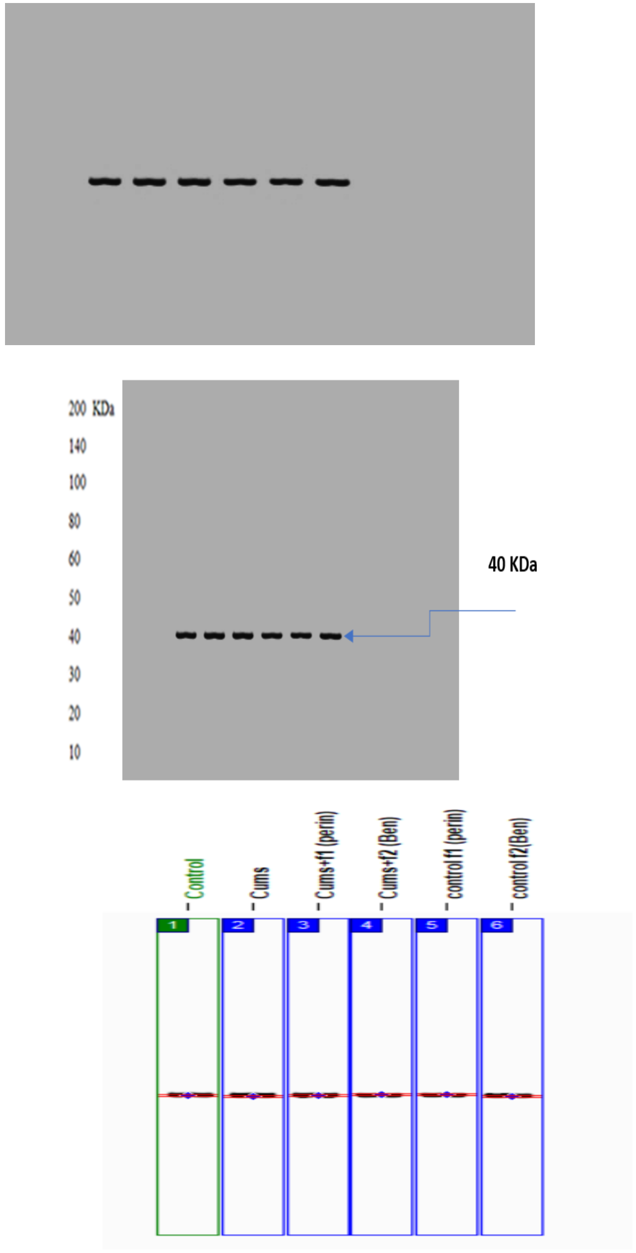

Frist Beta Actin 3 image for hippocampus

Beta Actin 1 (hippocampus)

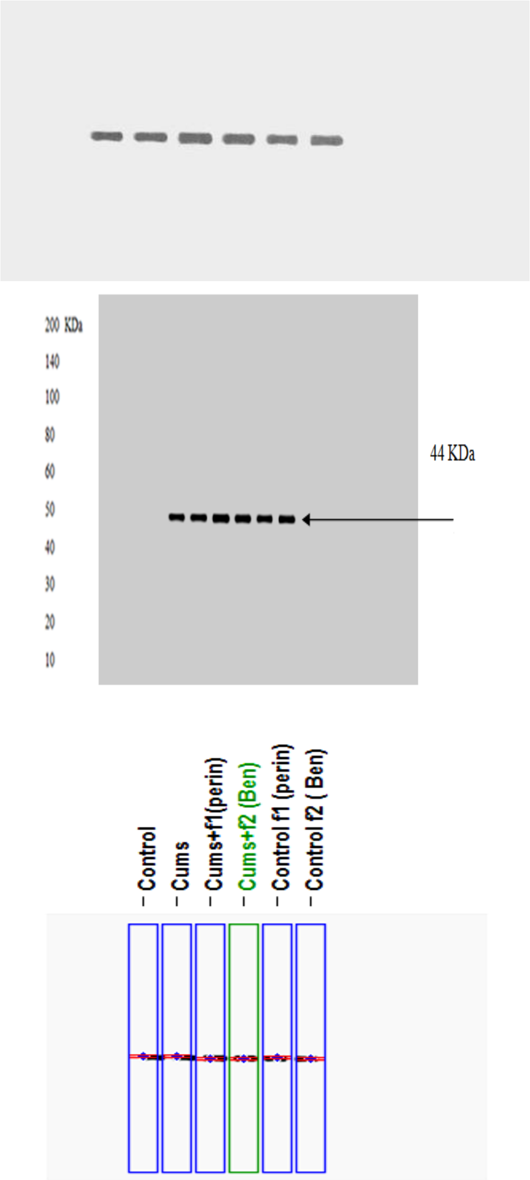

Beta Actin 2 (hippocampus)

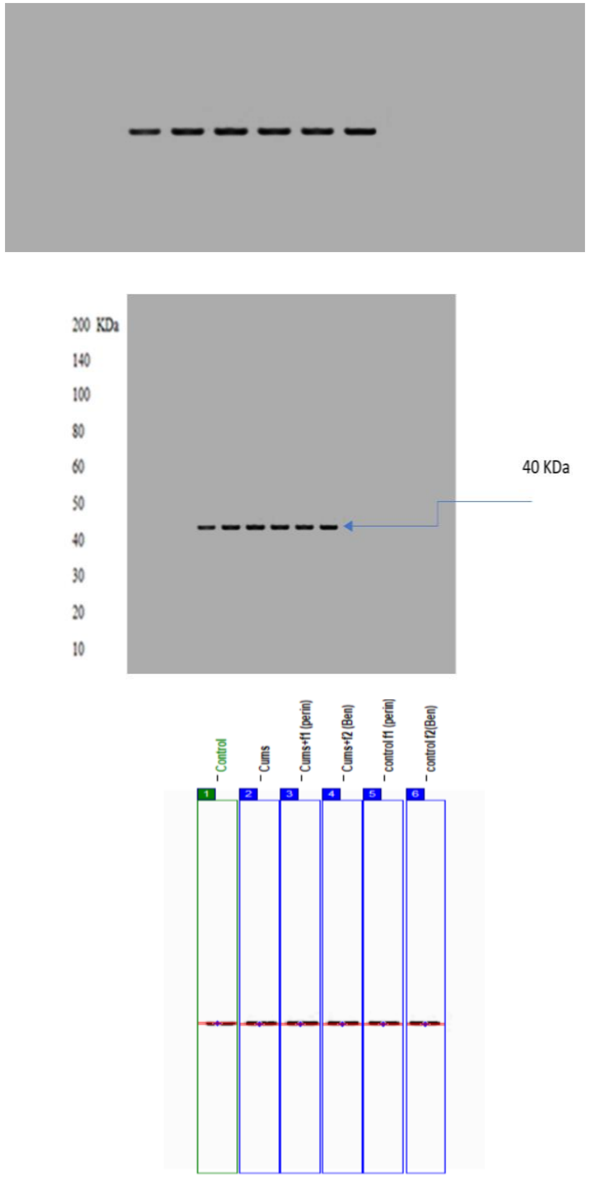

Beta Actin 3 (hippocampus)

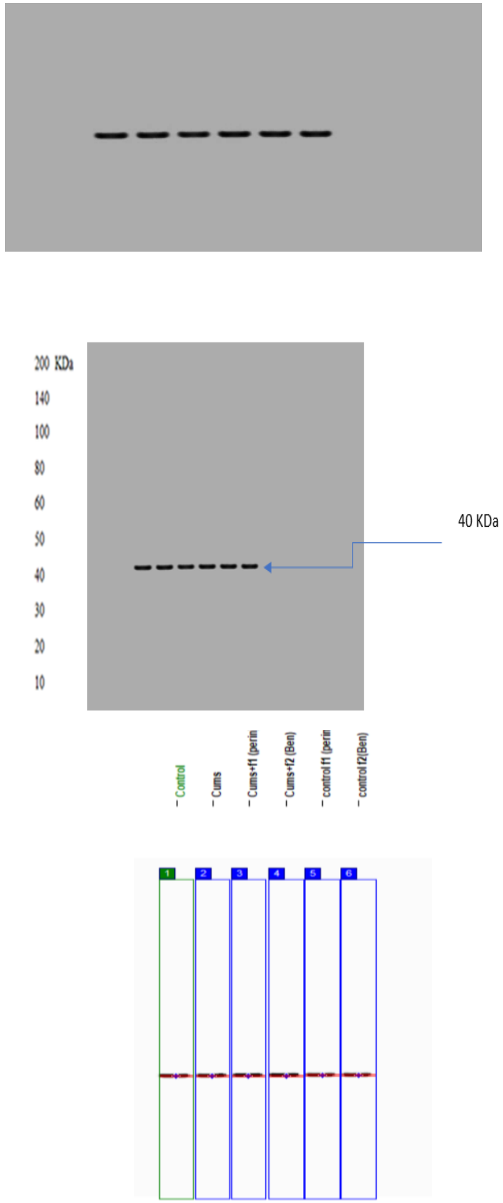

3 image of hippocampus Ag II

1

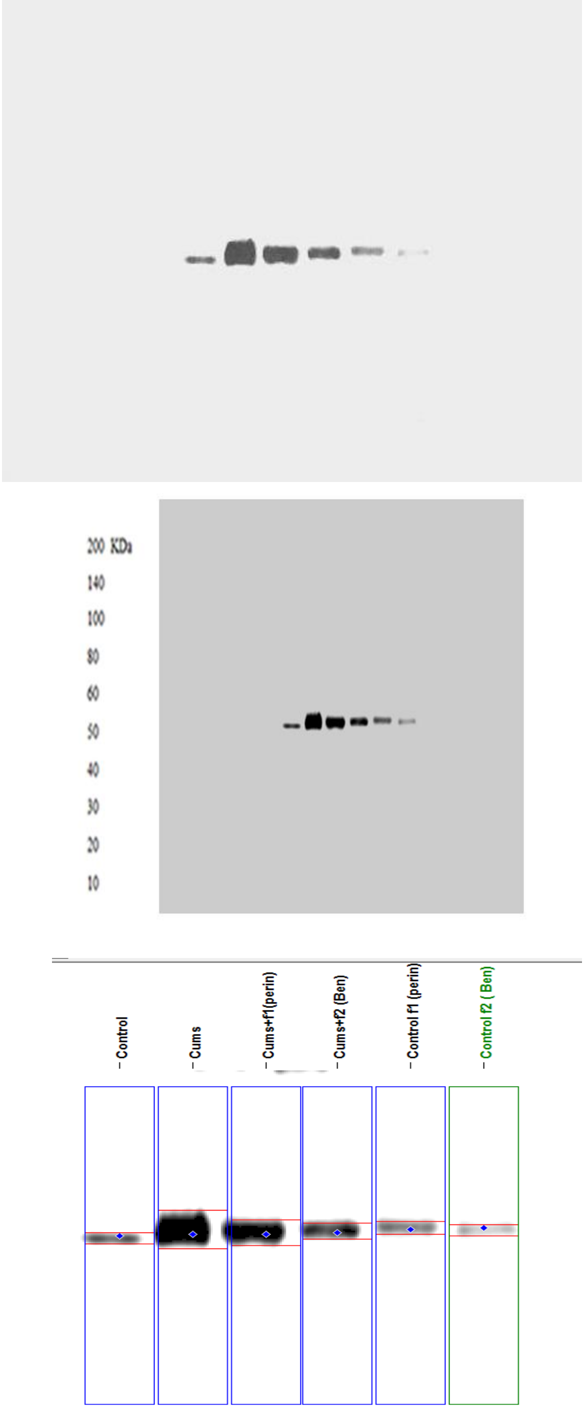

2

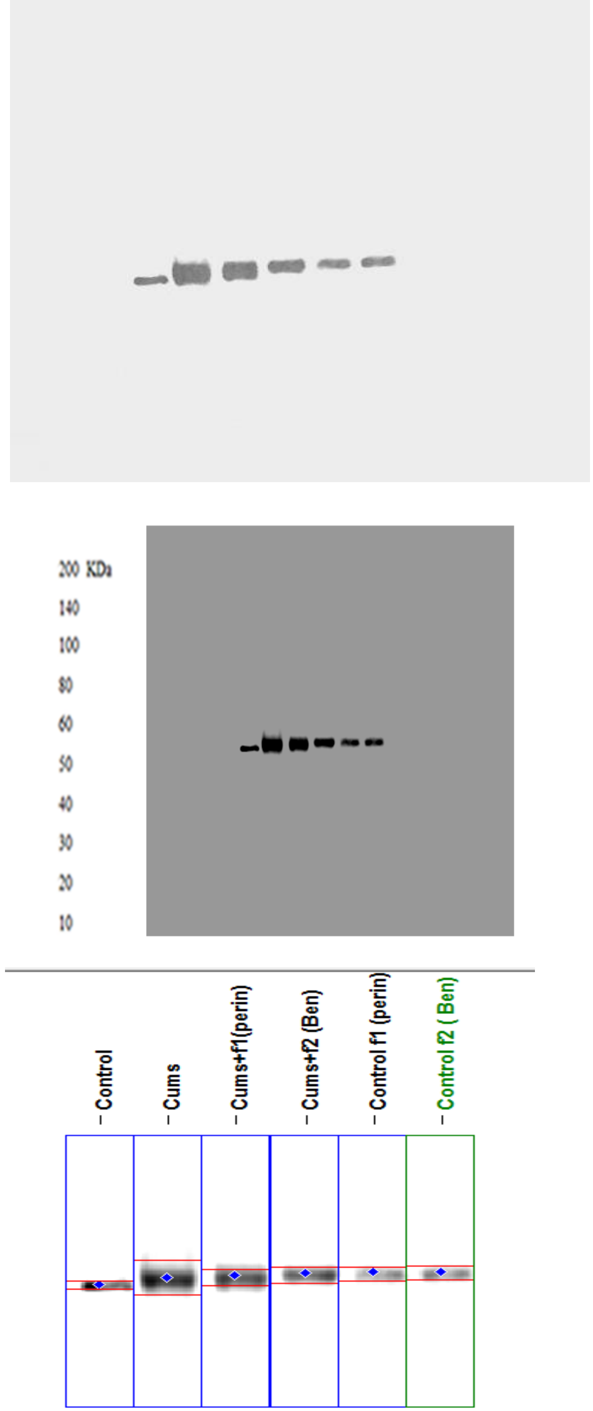

3

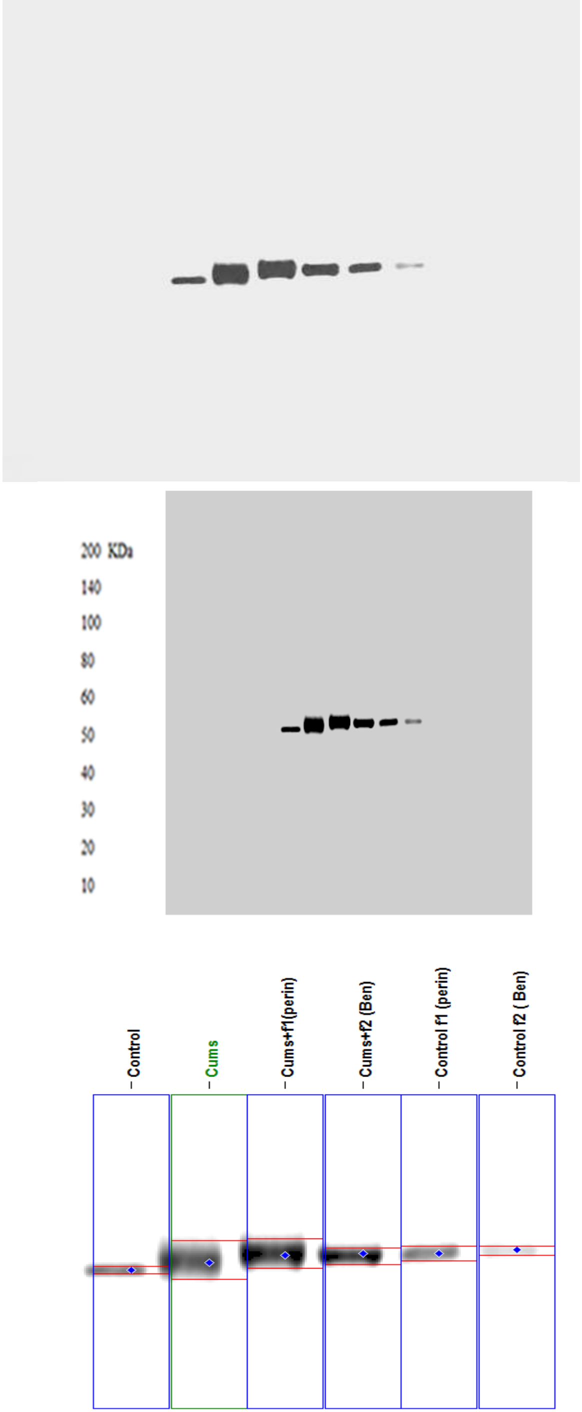

3 image of Cortex Ag II

1

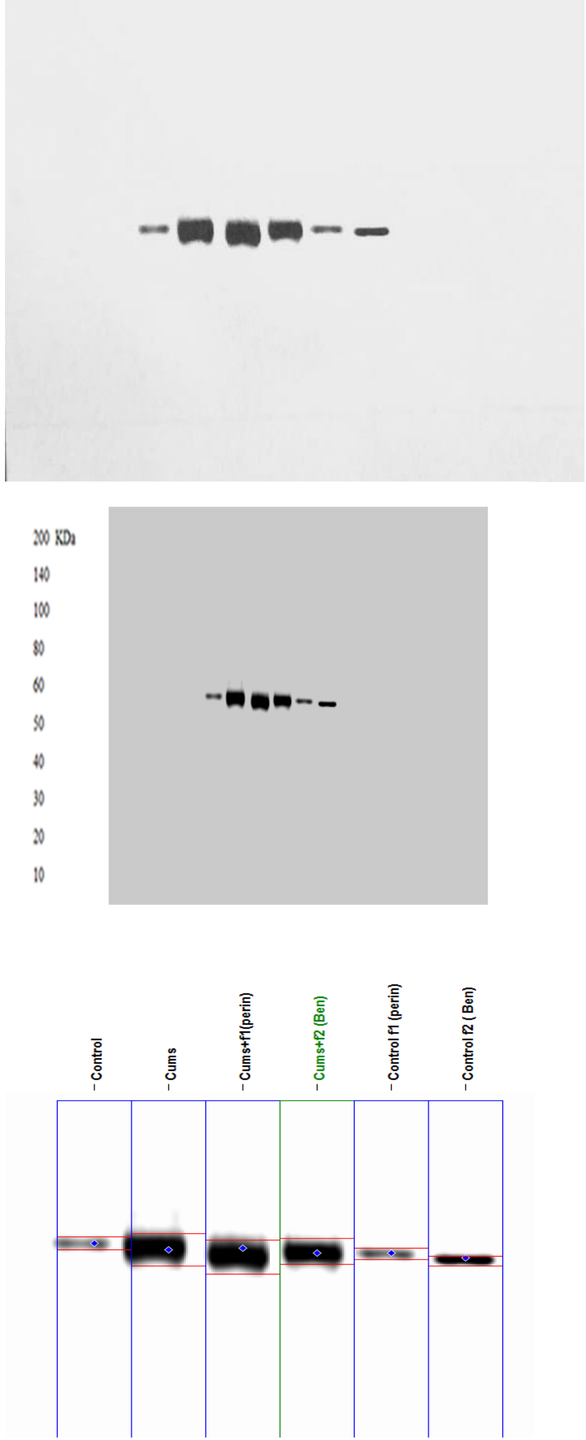

2

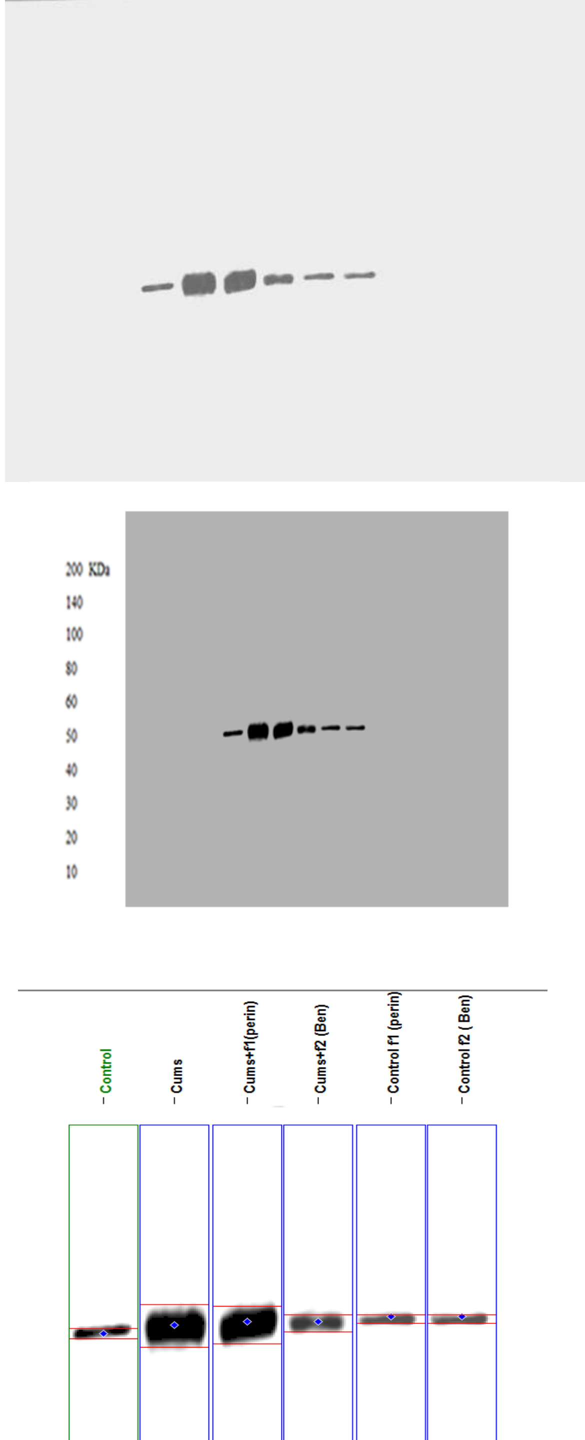

3

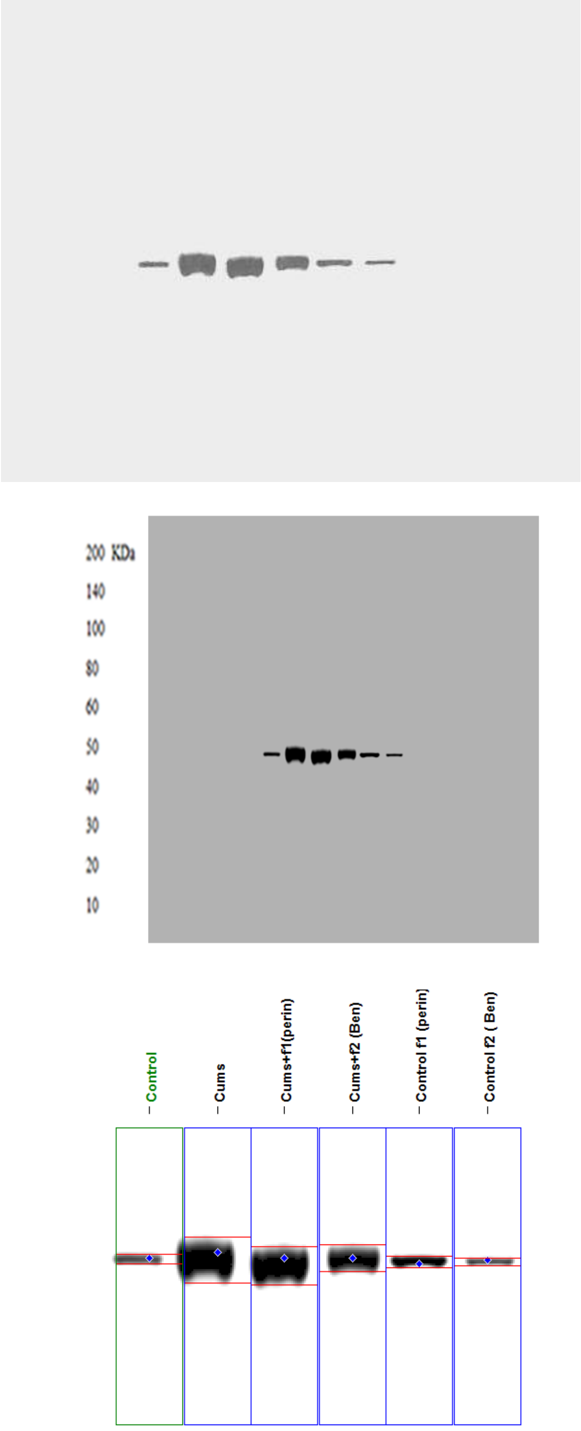

3 image hippocampus ATR2

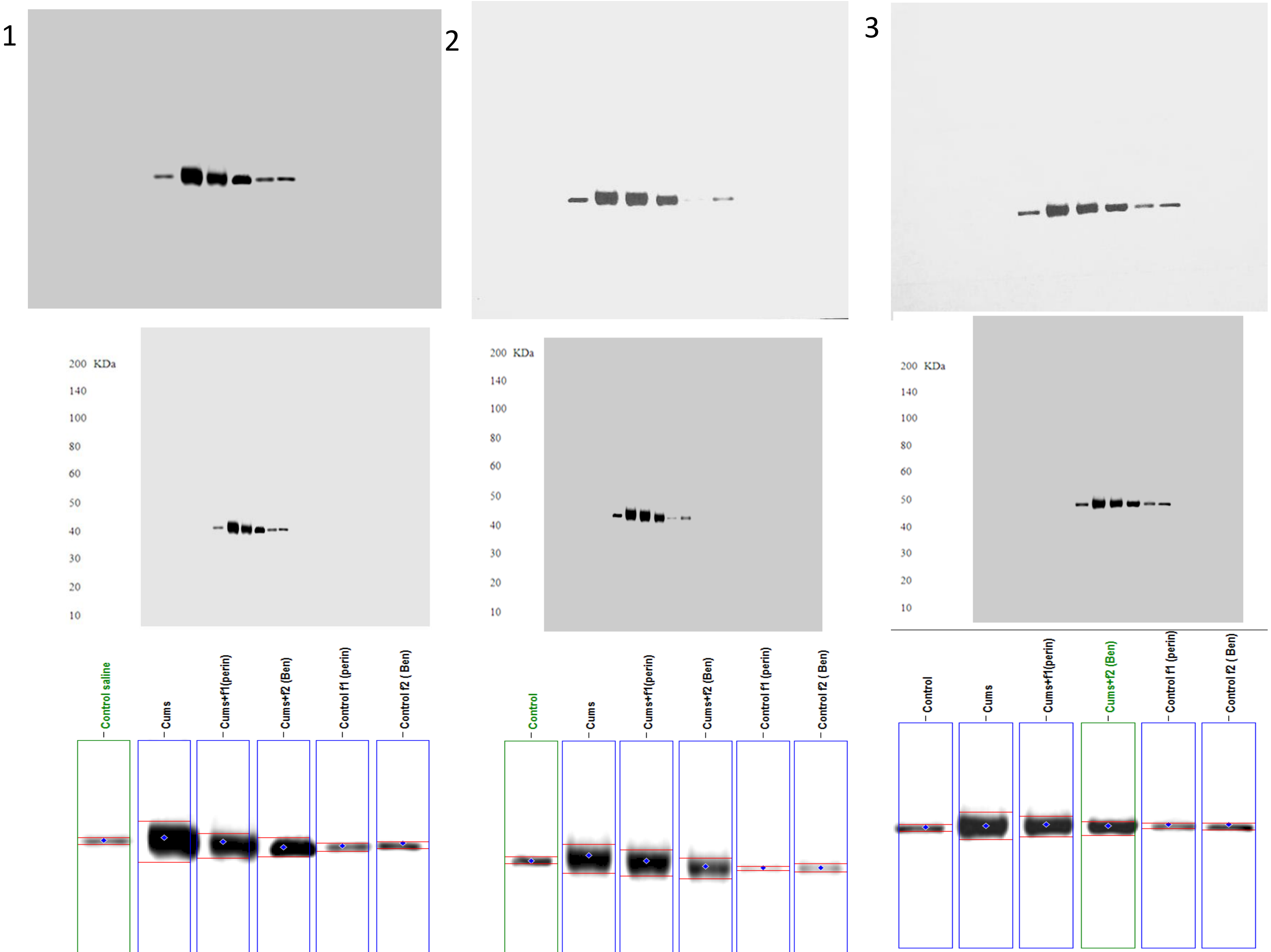

3 image Cortex ATR2

1

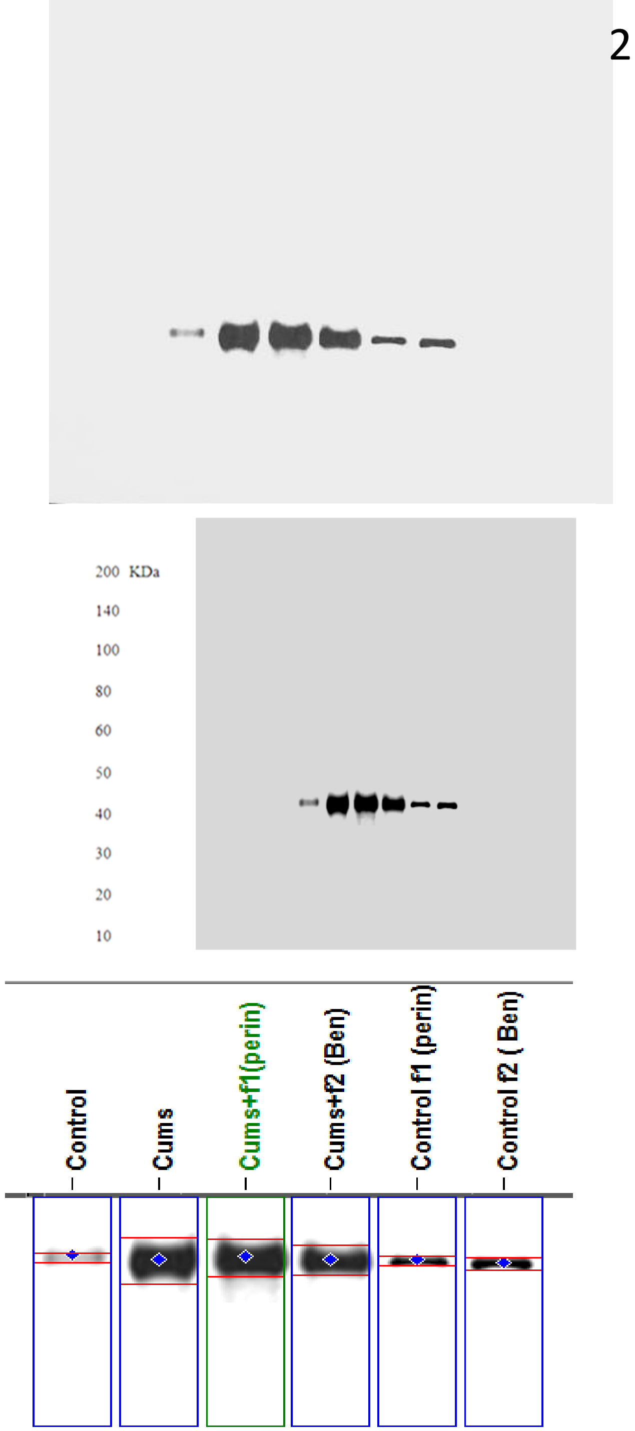

2

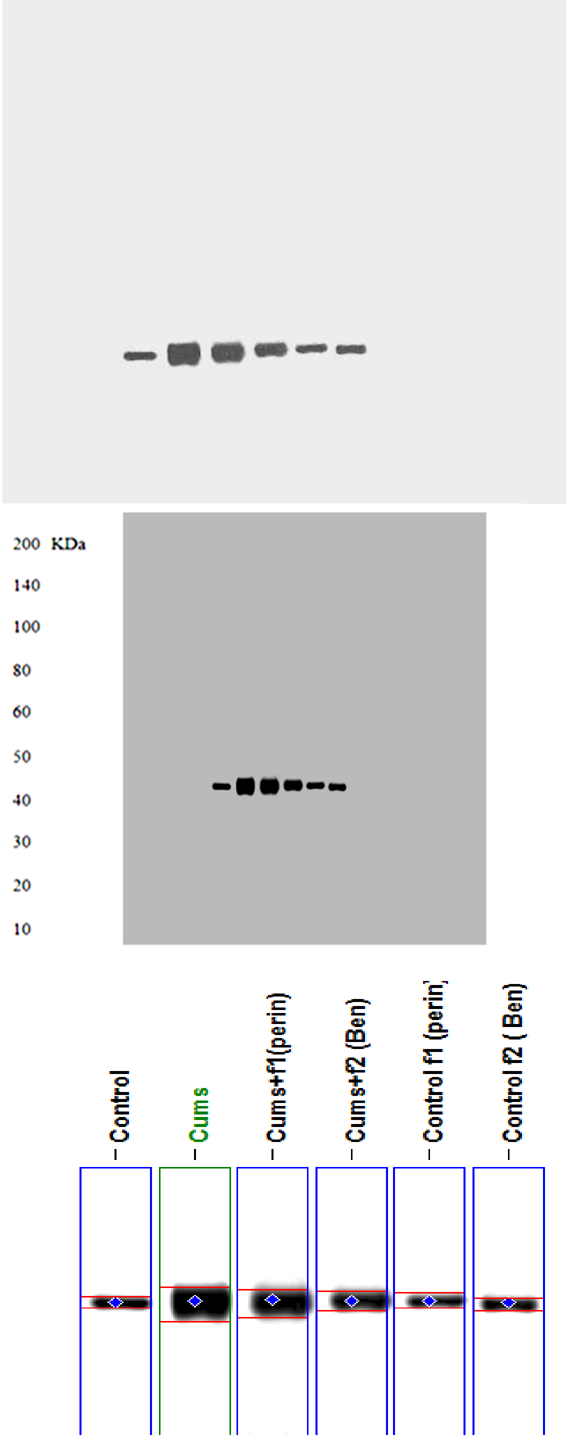

3

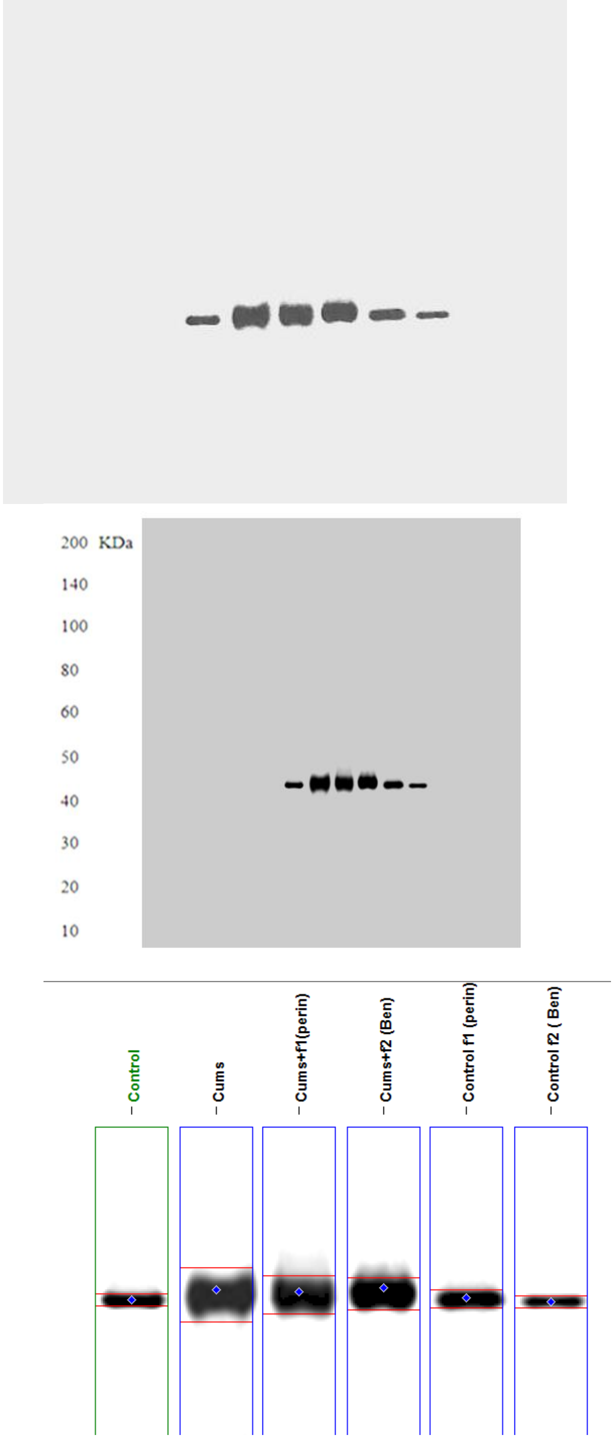

Supplement: Supplementary file 1 — (PDF 625 KB) [file 11481_2025_10244_MOESM1_ESM.pdf]
